# Recommended Tool Compounds: Thienotriazolodiazepines-Derivatized Chemical Probes to Target BET Bromodomains

**DOI:** 10.1021/acsptsci.4c00726

**Published:** 2025-03-14

**Authors:** Chuhui Huang, Kate S. Harris, Ghizal Siddiqui, Manuela Jörg

**Affiliations:** † Medicinal Chemistry, Monash Institute of Pharmaceutical Science, 2541 Monash University, 381 Royal Parade, Parkville, Victoria 3052, Australia; ‡ Chemistry-School of Natural and Environmental Sciences, 5994 Newcastle University, Newcastle Upon Tyne NE1 7RU, United Kingdom; § Drug Delivery, Disposition & Dynamics, Monash Institute of Pharmaceutical Science, 2541 Monash University, 381 Royal Parade, Parkville, Victoria 3052, Australia

**Keywords:** JQ1, bromodomain and extra-terminal domain (BET) family proteins, bromodomain-containing protein 4 (BRD4), chemical probes, protein degraders

## Abstract

Thienotriazolodiazepines, including (+)-JQ1 (**4**), are well-known inhibitors of the bromodomain (BD) and extra-terminal domain (BET) family of proteins. Despite the suboptimal physicochemical properties as a drug candidate, such as poor solubility and half-life, (+)-JQ1 (**4**) has proven as an effective chemical probe with high target potency and selectivity. (+)-JQ1 (**4**) and (+)-JQ1-derived chemical probes have played a vital role in chemical biology and drug discovery over the past decade, which is demonstrated by the high number of impactful research studies published since the disclosure of (+)-JQ1 (**4)** in 2010. In this review, we discuss the development of (+)-JQ1-derivatized chemical probes over the past decade and their significant contribution to scientific research. Specifically, we will summarize the development of innovative label-free and labeled (+)-JQ1-derivatized chemical probes, such as bivalent, covalent, and photoaffinity probes as well as protein degraders, with a focus on the design of these chemical probes.

## Graphical abstract

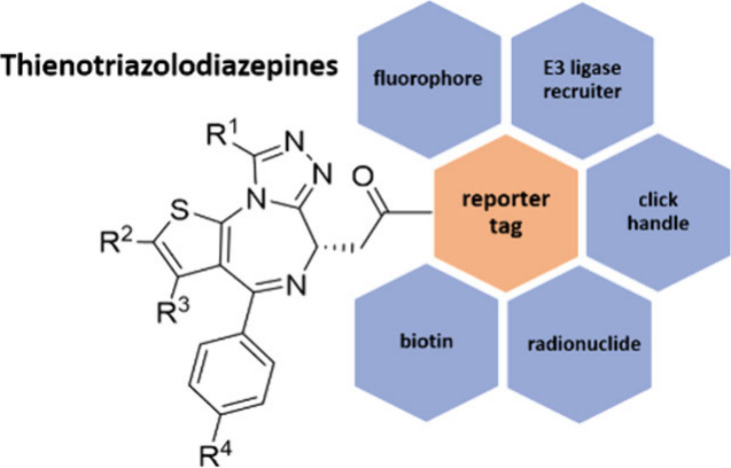

Thienotriazolodiazepines have a long history in drug discovery, dating back to the 1970s. Brotizolam^1^ (**1**) and etizolam^2^ (**2**) (Figure 1) were patented and approved in the 1980s for the treatment of insomnia and other psychological disorders such as anxiety.^1,2^ Both brotizolam and etizolam were reported to act by allosterically potentiating chloride currents induced by gamma-aminobutyric acid (GABA) in GABA_A_ receptors.^1,2^ In 2009, Mitsubishi Pharmaceuticals patented and reported that thienotriazolodiazepines were binding to bromodomain-containing protein 4 (BRD4).^3^ They revealed their discovery of thienotriazolodiazepines inhibitors, which displayed antitumor activity by binding between acetylated histone H3/H4 and a bromodomain (BD)-containing protein.^3^ One of their compounds, MS417 (**3**) (Figure 1), is still actively used in many studies today, including studies of human immunodeficiency virus (HIV)-associated kidney diseases and colorectal cancer.^4,5^

**Figure 1:**
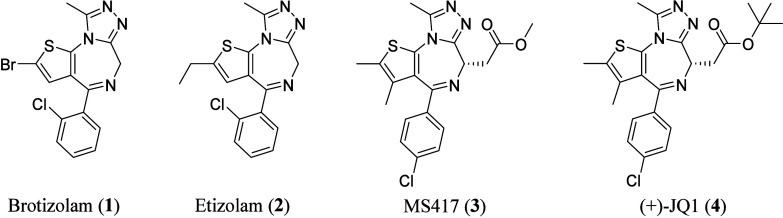
1st Generation of biologically active thienotriazolodiazepines inhibitors.

Bromodomains (BD) are acetyl-lysine-specific protein interaction modules present in proteins that have key roles in the regulation of gene transcription.^6^ The bromodomain and extra-terminal (BET) families are epigenetic readers that bind acetylated histones through BDs to regulate gene transcription.^6–8^ Members of the BET family have a common structure with two tandem BDs, namely bromodomain 1 (BD1) and bromodomain 2 (BD2).^8–10^ The BET family proteins, consisting of BRD2, BRD3, BRD4, and testis-specific bromodomain testis associated (BRDT), are major transcriptional regulators in biology that recruit transcription factors and coactivators to target gene sites, and activate ribonucleic acid (RNA) polymerase II machinery for transcriptional elongation.^11^ Recent advances of pharmacological inhibition of BRD4 protein with BD inhibitors has been developed as a promising therapeutic strategy for the treatment of cancers or solid tumors.^12^

In 2010, (6*S*)-4-(4-chlorophenyl)-2,3,9-trimethyl-6*H*-thieno­[3,2-*f*]­[1,2,4]­triazolo­[4,3-*a*]­[1,4]­diazepine-6-acetic acid 1,1-dimethylethyl ester (**4**), better known as (+)-JQ1 (Figure 1), was disclosed. Thienotriazolodiazepine derivative **4** was reported to have favorable synthetic accessibility and high selectivity for BD within the BET protein family, specifically (BRD2, BRD4, BRD3, BRDT), with half-maximum inhibitory concentrations (IC_50_) values of 77 nM and 33 nM, for BRD4­(BD1) and BRD4­(BD2), respectively.^13^ (+)-JQ1 (**4**) has since had a significant impact in pharmaceutical research and development due to the pioneering move by the Bradner group to freely distribute (+)-JQ1 (**4**) to the scientific community.^14,15^ As a result, over a thousand studies have been performed using (+)-JQ1 (**4**) to understand its pharmacological function in biological systems.^16^

The first report of (+)-JQ1 (**4**) by Bradner et al., successfully demonstrated the efficacy of (+)-JQ1 (**4**) on NUT-midline carcinoma (NMC) to attenuate protein–protein interactions and epigenetic targets by a small molecule.^13^ Furthermore, a cocrystal structure (PDB #3MXF)­(Figure 2) had shown that (+)-JQ1 (**4**) competitively binds to the acetyl-lysine recognition pocket, forming essential hydrogen bonds with key residues in the protein and displacing BDs, specifically BRD4, from chromatin, disrupting its biological function.^13,17^ Additional studies showed the potential of (+)-JQ1 (**4**) in downregulating myelocytomatosis oncogene (MYC) transcription.^18–25^ MYC proteins function as transcriptional modulators, regulating genes involved in several cellular processes such as cell growth, cell cycle, differentiation, apoptosis, angiogenesis, metabolism, deoxyribonucleic acid (DNA) repair, protein translation, immune response and stem cell formation.^26^ With (+)-JQ1 (**4**), BET BDs were proven to be pertinent in MYC transcription as the inhibition of BDs was shown to be modulating MYC pathogenesis,^18–25^ diminishing MYC mRNA to trigger G1 cell cycle arrest and apoptosis.^27,28^ (+)-JQ1’s antileukemic effects *in vitro* and *in vivo* via the suppression of BRD4, suggested (+)-JQ1 (**4**)’s pharmacological means to suppress MYC in cancer.^15–22^ As the pharmacologic inhibition of MYC function has proven challenging, the discovery of (+)-JQ1’s indirect regulation effects on MYC signified a breakthrough in cancer therapeutics.^27,28^

**Figure 2:**
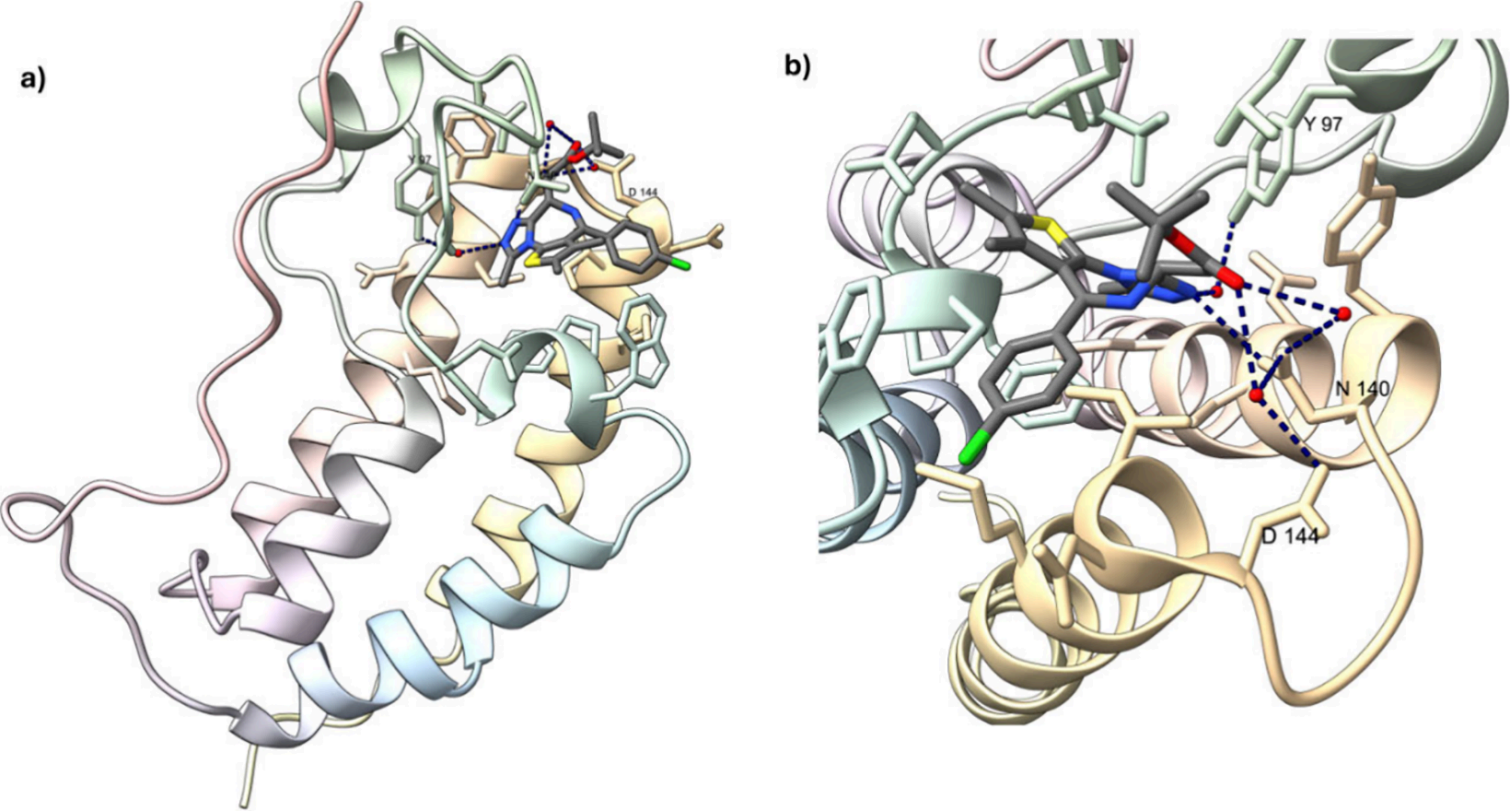
(a) The first bromodomain of human BDR4 in complex with inhibitor (+)-JQ1 (**4**) and (b) image focused on the acetyl-lysine recognition pocket, showing key interactions between BDR4 and (+)-JQ1 (**4**). Figure produced from PDB code 3MXF, using ChimeraX version 1.8.

Additional studies revealed that upregulation of BET proteins led to deregulation of transcriptional programmes, which had been linked to the development of several diseases such as cancer, inflammation, infectious diseases and hematological malignancies.^19,21,23^ Numerous studies have also been done to explore (+)-JQ1’s potential as a male contraceptive.^6,8,9,29,30^ More recently, the effect of (+)-JQ1 (**4**) on microglia in neurological disorders, such as glioblastoma^29^ and Alzheimer’s disease^31^ have been reported, further expanding the scope of potential indications to neurological disorders by targeting BET proteins.

The discovery of (+)-JQ1 (**4**) opened up the investigations on BET proteins, especially BRD4, as potential therapeutic targets in many diseases,^32^ particularly in oncology. This resulted in further studies in search of more efficacious and specific inhibitors of BDs of the BET family.^10,33^ Although, a number of (+)-JQ1-derived BET inhibitors have entered clinical trials, resistance or serious side effects have limited their progression into the clinic.^6,11,34^

This led to a trend of moving toward (+)-JQ1-derived modalities or combination therapy to overcome resistance and improve the effectiveness of current oncology treatments.^33,35–49^ Over the past decade, more novel (+)-JQ1-derived chemical probes have been developed to understand the biology of BET proteins and overcome the challenges of countering BET proteins in many diseases. In addition to key protein degraders which are currently trending, this review will discuss significant label-free and labeled (+)-JQ1-derivatized chemical probes that were developed.

## Label-Free Chemical Probes

Chemical probes have conventionally been defined as highly characterized small molecules that selectively bind to their biomolecular target, usually a protein, and help to elucidate and validate the targets’ role in biological systems using biochemical and cellular assays or in living animals.^50–54^ However, the presence of weak and nonselective probes has resulted in misleading and poor-quality results.^50,53^ Consequently, criteria have been set in consensus by the chemical biologists community to ensure the selection of high-quality probes.^51,53,55^ According to consensus criteria, chemical probes must be potent (IC_50_ or *K*_d_ < 100 nM in biochemical assays, EC_50_ < 1 μM in cellular assays) and selective (selectivity >30-fold within the protein target family, with extensive profiling of off-targets outside the protein target family).^50,51,53^ In addition, as discussed in the work of Bunnage and co-workers,^56^ chemical probes should be able to (1) reach the site of action at pharmacologically relevant concentrations, (2) display *in vitro* evidence of target engagement and selectivity, (3) provide sufficient data to assign phenotypic results to an original structure or a well characterized derivative, and (4) provide cellular activity data to answer a hypothesis on the role of the target.^51,56^

Many label-free JQ1-derivatized probes have been reported and tested to improve their pharmacological properties from those of the parent compound. The next section of this review highlights key label-free (+)-JQ1-derived chemical probes including thienotriazolodiazepine inhibitors and dual-targeting inhibitors. For a more comprehensive overview of BET inhibitors, encompassing the synthesis, design and the biological activities of (+)-JQ1 (**4**) analogs for cancer therapy, we refer readers to a recently published article by Laxmi et al.^57^

## Thienotriazolodiazepines Inhibitors

A number of thienotriazolodiazepine inhibitors were brought into human clinical studies, such as I-BET762 (**5**), TEN010 (**6**), and OTX015 (**7**) (Figure 3 and Table 1),^25,58,59^ displayed nanomolar potency against BET proteins, mainly on BRD2, BRD3, and BRD4.^33,60^ I-BET762 (**5**) (Figure 3), which contains an ethyl amine instead of butyl ester as the carbonyl substituent, was found to display higher affinity, efficacy and selectivity compared to (+)-JQ1 (**4**). Structure–activity relationship (SAR) studies demonstrated that having an electron-donating methoxy group on the *para*-position of the benzodiazepine scaffold increased the potency of the compound.^61^ Besides that, a chloro-substituent at the para-position on the phenyl ring substituent improved selectivity and prevented binding to the GABA receptor, while an acetamide substituent on the diazepine improved the stability.^61^

**Figure 3:**
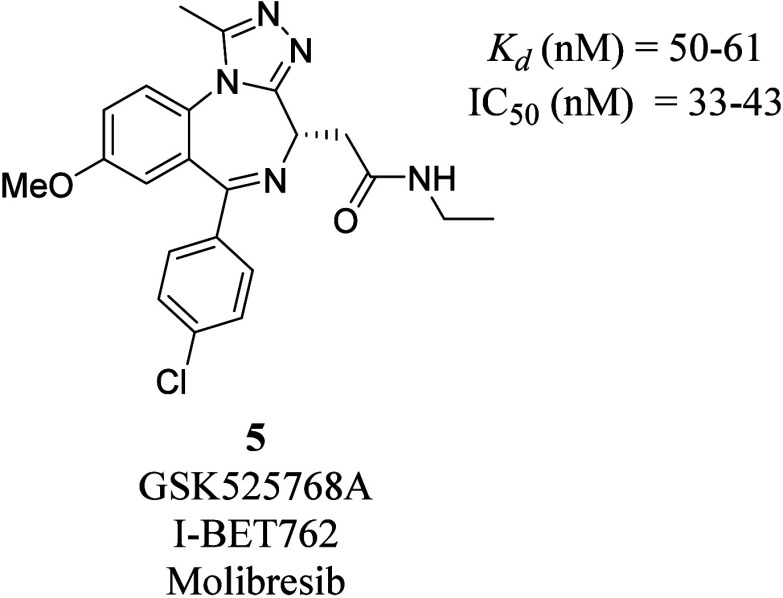
Chemical structure of the highly potent BRD4 inhibitor I-BET762 (**5**).

**Table 1:**
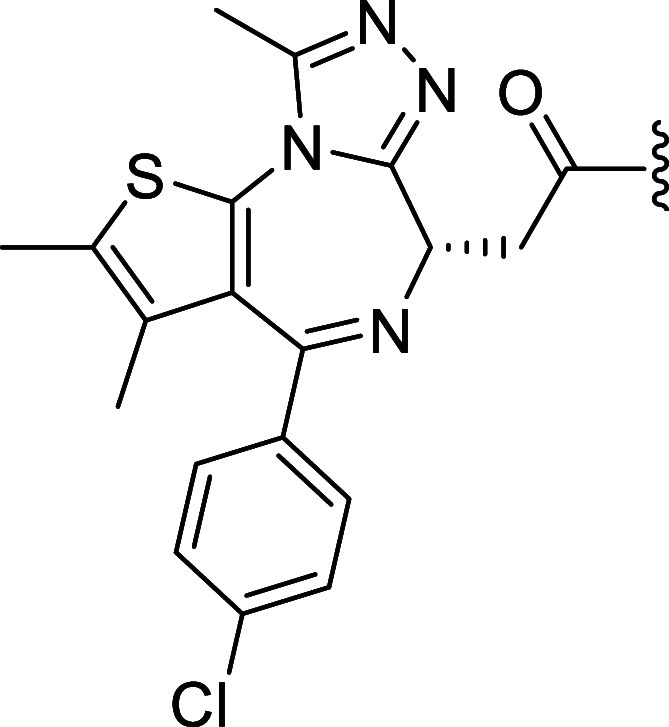

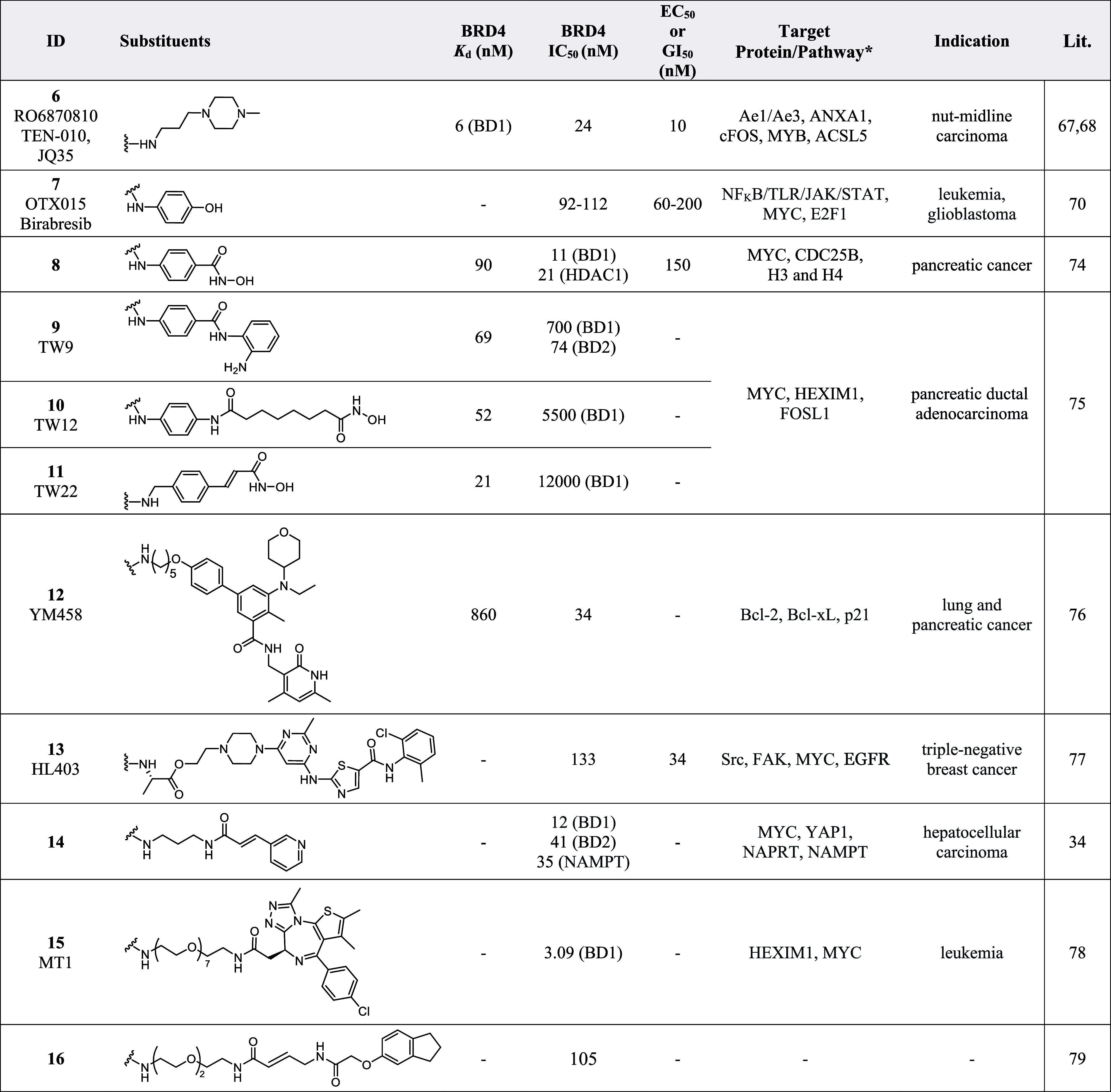
Label-Free (+)-JQ1-Derivatized Probes with Their Respective Potencies, Target Protein/Pathways, and Targeted Indications^a^

a Ae1/Ae3 = Anion exchanger 1/3, ANXA1= Annexin A1, cFOS = Fos proto-oncogene, MYB = Myeloblastosis, ACSL5 = Long-chain-fatty acid CoA ligase 5, NF_K_B = Nuclear factor kappa-light-chain-enhancer of activated B cells, TLR = Toll-like receptor, JAK = Janus kinase, STAT = Signal transducer and activator of transcription, E2F1 = E2F transcription factor 1, CDC25B = M-phase inducer phosphatase 2, H3 = Histone H3, H4 = Histone H4, HEXIM1 = Hexamethylene bis-acetamide-inducible protein 1, FOSL1 = FOS like 1, Bcl-2 = B-cell lymphoma 2, Bcl-xLl = B-cell lymphoma-extra large, p21 = Cyclin-dependent kinase inhibitor 1, YAP1 = Yes-associated protein 1, NAPRT/NAMPT = Nicotinate phosphoribosyltransferase. *Non-BET enriched proteins.

Analogues of (−)-JQ1, the *R*-stereochemistry of JQ1, served as a quality negative control of the active enantiomer (+)-JQ1 (**4**) displaying no inhibition of BRD1–4 proteins.^13^ For instance, the (*S*)-enantiomer of **5** was found to be the active isomer,^61^ which exhibited no interaction with other BD-containing proteins from each arm of the phylogeny tree, and was selective against a panel of 38 unrelated proteins.^61,62^ Whereas, the *R*-enantiomer of **5** was inactive against BET proteins and, therefore was considered as a suitable negative control.^62^ A crystal structure of **5** binding to BD1 PDB # (3P5O) revealed the binding position of **5** at the acetyl-lysine binding pocket.^61,62^ Biological studies elucidated that **5** was able to disrupt chromatin complexes responsible for the expression of key inflammatory genes in activated macrophages by interfering with the recognition of acetylated histones by BET proteins.^61,62^ Displaying good bioavailability, **5** advanced into clinical development for the treatment of NMC and small-cell lung, castration-resistant prostate, triple-negative breast, and gastrointestinal stromal cancer.^61,63–65^ Recently, **5** was also found to possess antidiabetic effect in mice models,^66^ signifying additional disease treatment potential. Nonetheless, adverse effects such as thrombocytopenia, nausea, and decreased appetite were observed for **5** in clinical trials for cancer, suggesting it may share the unfavorable toxicity profile with other earlier-generation BET inhibitors.^25,63^

RO6870810 (**6**) (formerly known as TEN-010 and (+)-JQ35) (Table 1), a thienotriazolodiazepine inhibitor patented in 2006 by Tensha Therapeutics, was discovered as a second-generation inhibitor of (+)-JQ1 (**4**).^67^ Comprising of a 3-(4-methylpiperazin-1-yl)­propan-1-amine substituent at the carbonyl position, **6** displayed improved solubility and *in vitro* potency for NMC.^67^ Inhibitor **6** was in clinical trials for acute myeloid leukemia, myelodysplastic syndrome, and other solid cancers.^25,68^ Initial clinical data suggested that **6** possessed more favorable pharmacokinetic properties than (+)-JQ1 (**4**), including higher time to peak (*t*_max_) and longer half-life (*t*_1/2_).^25,68^ However, some common adverse effects such as fatigue, injection site reactions, diarrhea, loss of appetite, and nausea were also observed in clinical trials.^69^

The mechanism of BET inhibitor OTX015 (**7**) (Birabresib, MK-8628) (Table 1) was studied in a panel of 33 cell lines derived from mature B-cell lymphoid tumors.^70^ Inhibitor **7** was shown to be active in a dose-dependent manner in almost all the tested cell lines.^70^ Many other studies conducted on **7** also revealed potential in inhibiting SARS-CoV-2 infection^71^ and HIV.^72^ Promising results from the preclinical evaluation of **7** in multiple myeloma led to elevated expectations for achieving clinical responses.^33^ Unfortunately, patients with multiple myeloma were not responsive to treatment with **7** in the first clinical trial, and almost all of the patients displayed dose-limiting toxicities, including thrombocytopenia, anemia, neutropenia, gastrointestinal events and fatigue.^33^

## Dual and Bivalent Inhibitors

Dual-targeting inhibitors have been of increasing interest in recent years, due to their ability to present an effective alternative approach to drug combinations.^73^ Usually comprising of two well-developed inhibitors with predefined biological profiles, these dual-targeting inhibitors are beneficial for addressing limited efficiencies, poor safety and resistant profiles of an individual drug.^73^

It is noteworthy that most thienotriazolodiazepine inhibitors differ only in the substituent that is installed on the carbonyl group, highlighting the importance of the thienotriazolodiazepine pharmacophore for binding to BET family proteins. In agreement with this trend, (+)-JQ1-derived dual inhibitors usually have the second active inhibitor installed at the carbonyl position of (+)-JQ1 (**4**).

The first (+)-JQ1-derived dual inhibitor **8** (Table 1) simultaneously targeted BET and histone deacetylase (HDAC) for the treatment of pancreatic cancer.^74^ It comprised of (+)-JQ1 (**4**) and the HDAC inhibitor vorinostat, and molecular docking study demonstrated successful binding of **8** with both BRD4 and HDAC1.^74^ Dual inhibitor **8** had better *in vitro* potency against pancreatic adenocarcinoma Capan-1 cells and *in vivo* antitumor potency than (+)-JQ1 (**4**) and vorinostat, either alone or and in combination, highlighting the advantages of BET/HDAC dual inhibitors for more effective treatment of pancreatic cancer.^74^ Dual BET/HDAC inhibitors TW9, TW12 and TW22 (**9**–**11**) (Table 1) comprising of (+)-JQ1 (**4**) and clinical HDAC inhibitors, tacedinaline, vorinostat, and panobinostat, respectively, were shown to be effective for the treatment of pancreatic ductal adenocarcinoma.^75^

YM458 (**12**) (Table 1) is a novel dual inhibitor of enhancer of zeste homologue 2 (EZH2)/BRD4, which consists of EZH2 inhibitor EPZ6438 linked to (+)-JQ1 (**4**).^76^ Despite having higher molecular weight, dual inhibitor **12** exhibited better antiproliferative activity than EZH2 inhibitors or EPZ6438 alone against a series of solid cancer cell lines, including pancreatic, colon, and lung cancers.^76^ The aforementioned results suggest that dual BRD4/EZH2 inhibitors may be utilized as a new class of targeted therapy against a wide range of solid tumors.^76^

The synergistic effect of combination therapy using nonreceptor tyrosine kinase (SRC) inhibitor dasatinib and (+)-JQ1 (**4**) on JQ1-resistant triple-negative breast cancer (TNBC) cell line SUM159R led to the development of a class of dual BRD4/SRC inhibitors.^77^ The SAR studies revealed that longer and rigid linkers, such as PEG, aromatic heterocycles and cyclic alkanes, were found to reduce potency, while substituted amino acid linkers, such as valine and alanine, displayed better inhibitory activity.^77^ By effectively optimizing the linker between the two inhibitors, HL403 (**13**) (Table 1) with a 3-carbon linker, displayed efficacy against TNBC both *in vitro* and *in vivo*, and successfully suppressed the migration and invasion of TNBC cells.^77^

A recent combination study that utilized (+)-JQ1 (**4**) with FK866, a highly specific noncompetitive inhibitor of nicotinamide phosphoribosyltransferase (NAMPT), demonstrated superior antiproliferative activity on a hepatocellular carcinoma (HCC) cell line, compared to monotherapy with (+)-JQ1 (**4**) or FK866.^34^ These results led to the rational design of a BET/NAMPT dual inhibitor **14** (Table 1) based on molecular dynamics studies, which combined (+)-JQ1 with the (4-aminophenyl)­ethylamine active motif in FK866.^34^ Different linkers were investigated to conjugate the active ligands, and it was found that flexible alkyl chain linkers performed better than rigid piperidinyl- and phenyl-based linkers.^34^ The length of the alkyl chain greatly affected the efficacy of the inhibitors, with shorter linker lengths displaying higher inhibition of BRD4 and lower inhibition of NAMPT protein, while increasing the linker length led to lower BRD4 inhibition and higher NAMPT inhibition.^34^ These results demonstrated the importance of linker optimization in bifunctional molecules, as length and flexibility affected the potency of the molecules.^34^ Compound **14**, with a 3-carbon linker, was found to display the best potency with a balanced effect on the inhibition of both NAMPT and BRD4.^34^ It was also shown to inhibit the growth of HCC cells by reducing nicotinamide adenine dinucleotide levels and downregulating the expression of genes that promoted tumor development.^34^ *In vivo* studies showed that **14** displayed effective suppression of tumor proliferation without significant toxic effects, thus presenting itself as the first efficient BRD4/NAMPT dual-inhibitor for the treatment of HCC.^34^

MT1 (**15**) (Table 1) is a bivalent chemical probe of BET proteins, consisting of two (+)-JQ1 (**4**) ligands that are connected via a linker with 7-units of polyethylene glycol (PEG).^78^ Despite the high molecular weight, **15** displayed better pharmacokinetics and selectivity compared to (+)-JQ1 (**4**),^25^ with 7-fold higher potency (IC_50_ of 3.09 nM), and a 100-fold increase in cellular potency (IC_50_ of 0.17 nM and 0.792 nM) in acute monocytic leukemia MV4-11 and NMC797 cells, respectively.^78^ The bivalent chemical probe **15** also downregulated MYC in MV4-11 cells.^78^ In brief, **15** was identified as an efficient probe for BET proteins, which provided the rationale for further development of multidomain inhibitors of epigenetic reader proteins.^78^

Probe **16** (Table 1), a novel covalently binding bivalent (+)-JQ1-derivatized chemical probe, was designed using both fragment-based drug discovery and computational chemistry.^79^ (+)-JQ1 (**4**) was linked to a cysteine selective fragment using 2-units of polyethylene glycol PEG linker.^79^ The cysteine selective fragment, which contains a cysteine-reactive Michael acceptor, was identified in a library screen showing selective covalent binding of BRD4­(BD2) relative to other BET proteins.^79^ Crystallography and nuclear magnetic resonance studies with **16** successfully identified a novel orthogonal Cys356 binding site within BRD4, which is specific to BRD4 compared to other BET proteins, allowing potential probing of acetylation-independent functions of BD-containing proteins and alternative means of achieving selectivity for BRD4.^79^

## Labeled Chemical Probes

Labeled chemical probes are bioactive molecules that are derivatized with reporter tags or ligation handles and have emerged as powerful tools to investigate and characterize ligand–protein interactions *in vitro* and *in vivo*.^80,81^ By functionalizing the bioactive pharmacophore with fluorescent tags or radioligands, the probe–protein complex formed can then be detected via localization imaging of the compound.^80^ Fluorophores such as fluorescein and rhodamine, and radioisotope fluorine-18 are common tags used for visualization through confocal microscopy or positron emission tomography (PET) scans in *in vivo* biological studies.^80^ Biotinylation is another common method to attach biotin to targeted protein, which will in turn bind noncovalently with streptavidin or avidin conjugates for affinity pulldown experiments or visualization.^80^ Streptavidin conjugated to magnetic beads or horseradish peroxidase can be utilized to isolate target protein for further analysis such as immunoblotting and/or proteomics.^80^

Ligation handles are functionalities that are installed for performing biorthogonal reactions which are usually fast and compatible under physiological and aqueous conditions, without interfering with biological processes and affecting the ligands’ potency and selectivity.^80^ Examples of commonly utilized biorthogonal reactions in biological studies are Staudinger ligation between azide and phosphine, copper-catalyzed azide–alkyne cycloaddition (CuAAC), strain-promoted azide–alkyne cycloaddition (SPAAC), and inverse electron-demand Diels–Alder (IEDDA) cycloadditions of azide and *trans*-cyclooctene (TCO).^82^ Click reactions between functionalized ligands and reporters with orthogonal affinity allow capturing and labeling of target proteins for further studies to identify target protein/s, or to visualize the cellular localization of the compound, via immunoblot and proteomics analysis, or confocal microscopy, respectively.^80^

## Fluorescent Probes

Fluorescent probe (+)-JQ1-FITC **17** (Table 2) was developed and utilized to validate a novel time-resolved Förster resonance energy transfer (TR-FRET)-based platform that profiles cellular action of proteolysis targeting chimeras (PROTACs) in a high-throughput format.^83^ The fluorescein-containing probe **17** binds with high affinity to BRD4­(BD1) and BRD4­(BD2) (*K*_d_ values are 6.5 nM and 5.8 nM, respectively) and has an excitation and emission maxima (λ) of 495 and 525 nm, respectively.^83^ Employing single-domain nanobodies (nanosecondaries) labeled with CoraFluor-1, a TR-FRET donor complex with excellent stability and photophysical properties, this platform was shown to accurately quantify BRD4 protein levels in whole cell lysates and measure target engagement.^83^ Recently, probe **17** has also been reported in a protocol for the comprehensive biochemical and cellular profiling of small-molecule degraders using the CoraFluor TR-FRET technology.^84^

**Table 2:**
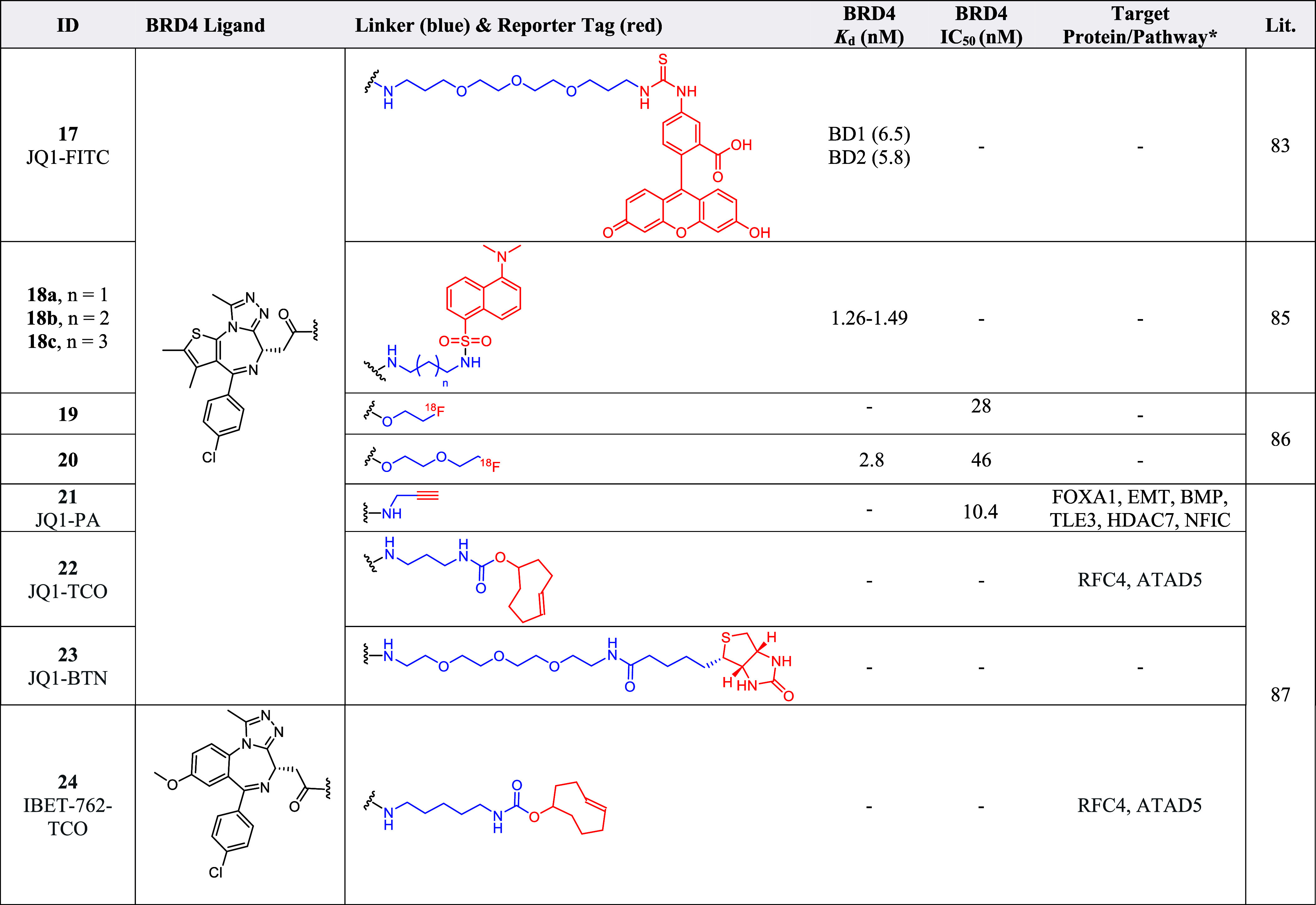
List of Reversible Labeled JQ1-Derivatized Probes^a^

a The blue portion represents the linker while the red portion represents reporter tag/click handle of the probes. TAD5 = ATPase family AAA domain containing 5, BMP = Bone morphogenetic proteins, EMT = Epithelial–mesenchymal transition, FOXA1 = Forkhead box protein A1, HDAC7 = Histone deacetylase 7, NFIC = Nuclear factor I C, RFC4 = Replication factor C subunit 4, TLE3 = Transducin-like enhancer protein 3. *Non-BET enriched proteins.

Environment-sensitive fluorescent probes **18a**–**c** (Table 2) were designed by tethering a dansyl amide fluorophore to (+)-JQ1 (**4**) via alkyl linkers of varying length.^85^ Probes **18a**–**c** possessed respectable Stoke shifts, with excitation wavelengths ranging between 350 and 365 nm and emission wavelengths of 495–500 nm.^85^ The fluorescence intensity of **18a**–**c** was also shown to decrease with solvent polarity, demonstrating environmental sensitivity in their fluorescence emission.^85^ **18a**–**c** were used to label and study the distribution of the BET family proteins in tumor cells and tissues, with **18a**, which contains the shortest alkyl linker, capable of distinguishing between tumor and normal tissues.^85^ In comparison to **17**, probe **18a** was tested directly on cells and tissues, rendering it suitable for immunofluorescent and diagnostic tools studies for cell and tissue staining as well as pathological and physiological studies of BET family proteins.^85^

## Positron Emission Tomography (PET) Radiotracers

PET radiotracers possess radioactivity that allows them to be monitored via PET scanning in *in vivo* or *in vitro* experiments.^80^ (+)-JQ1-based PET radiotracers containing a fluorine-18 radioisotope positioned at the final carbon atom of alkyl and PEG chains of different lengths were investigated.^86^ Tracers **19** and **20** (Table 2) were found to possess favorable BET proteins inhibitory affinity, selectivity, stability and also displayed blood–brain barrier permeability.^86^ Furthermore, due to the good brain uptake and binding specificity displayed in mice brains, **19** and **20**, could be used for BET protein imaging in the brain.^86^

## Ligation Handle Derivatives

Derivatives of BET inhibitors (+)-JQ1 (**4**) and IBET-762 (**5**) were synthesized with different ligation handles to obtain probes JQ1-propargyl amide (JQ1-PA) (**21**), JQ1-*trans*-cyclooctene (JQ1-TCO) (**22**), JQ1-biotin (JQ1-BTN) (**23**), and IBET-762-TCO (**24**) (Table 2).^87^ Fluorescent imaging reporter 488-azide and Cy5-tetrazine were used to perform bioorthogonal reactions with probes **21**–**24** for fluorescence microscopy and flow cytometry analysis, which successfully demonstrated a clear colocalization of the probes with the BRD4 protein in the cell nuclei.^87^ The fluorescent cell population was also successfully determined via fluorescence-activated cell sorting, demonstrating the use of labeled chemical probes with bioorthogonal reactive groups as an approach to assess intracellular target-probe colocalization, performing direct target engagement and measuring target occupancy in cells.^80,87^ Click proteomics was also performed to identify proteins captured from the lysate of K562 cells by click-probes **22** and **24** in the presence or absence of the respective competitor (IBET-151 and (+)-JQ1 (**4**)).^87^ The obtained results showed similarities in enrichment of BRD2, BRD3, BRD4 and also several proteins including replication factor C subunit 4 and ATPase family AAA domain containing 5 (ATAD5), denoting the identical cellular and molecular activity of these compounds and indicating the possibility of cross resistance prevalence.^87^

## Covalent Labeling

Covalent chemical probes contain reactive moieties that can form covalent bonds with protein targets.^88^ These probes can be beneficial for studying protein targets in a complex cellular environment for drug discovery biology and target validation programmes due to irreversible attachment to the protein target, increased duration of action and potentially improved selectivity for the target protein.^80^

With the trending interest in covalent probes, Workman and co-workers have proposed quality criteria for such emerging probes, adding to the criteria previously set for small molecular probes, such as potency of EC_50_ < 1 μM in cellular assays and selective (selectivity >30-fold within the protein target family, with extensive profiling of off-targets outside the protein target family).^54^ In addition, proteome-wide selectivity was also suggested to be assessed through unbiased methods such as MS-proteomics studies and pull-down experiments, utilizing bioorthogonal reactions as those mentioned in the previous section.^54^ A number of labeling methods have also been developed to capture protein targets via covalent bond formation, such as photoaffinity, photocatalysis, proximity and electroaffinity labeling methodologies for target deconvolution.^89–95^

The availability of a wider variety of reactive motifs will support target elucidation, both by providing additional tools for the capture of protein targets as well as alternative synthetic approaches for a given probe of interest.^89^ Ideally, the novel reactive motifs cause minimal cellular and protein damage during activation, rapidly generating short-lived reactive intermediates capable of residue-agnostic cross-linking, and minimally perturbing the native substrate–protein interactions.^89^

### Photoaffinity Labeling

In photoaffinity labeling (PAL), photoaffinity probes consist of photoreactive groups such as diazirines,^96^ aryl azides,^97^ or diaryl ketones^98^ that when exposed to ultraviolet (UV) light, form carbenes, nitrenes, or ketyl radicals,^89^ respectively. These high-energy intermediates are then capable of binding covalently to nearby proteins, giving the advantage of confining the adduct formation to the desired site and reducing nonspecific labeling compared to covalent probes with chemoreactive groups.^89^

Novel BRD4 targeting chemical probes **25** and **26** (Table 3) with diazirine linker and cyclopropene moieties were developed to enable copper-free bioorthogonal chemistry for target identification studies.^90^ In comparison to utilizing bulky PAL and click chemistry functionalities, such as benzophenone and TCO, respectively, the usage of small diazirine and cyclopropene functional groups present advantages of reduced steric hindrance to increase the probability of capturing protein targets via formation of covalent bonds.^90^ A series of probes **27**–**31** (Table 3), with a variety of bioorthogonal tags, such as alkenes, azides and tetrazines demonstrated positive target labeling, except **29**, which consist of a less reactive alkene group.^91^ The comparison studies of these probes revealed that (1) for proteomic profiling, in which click chemistry is carried out post-UV irradiation under *in vitro* conditions, terminal alkyne- and azide-containing photo-cross-linkers such as **27** and **30** were ideal due to their small size and minimum background labeling^91^ and (2) for live-cell imaging, photo-cross-linkers with a suitable cyclopropene, azide or tetrazine (i.e., in **26**, **30** and **31**) may be used to provide additional information on drug–target interaction at subcellular levels.^91^ (3) In cases in which both live-cell imaging and proteomic profiling were carried out, azide-containing probes were the most ideal (i.e., **30**), as they afford the best compromise in size, stability, and reactivity in biological environments without side reactions toward endogenous functional groups.^91^

**Table 3:**
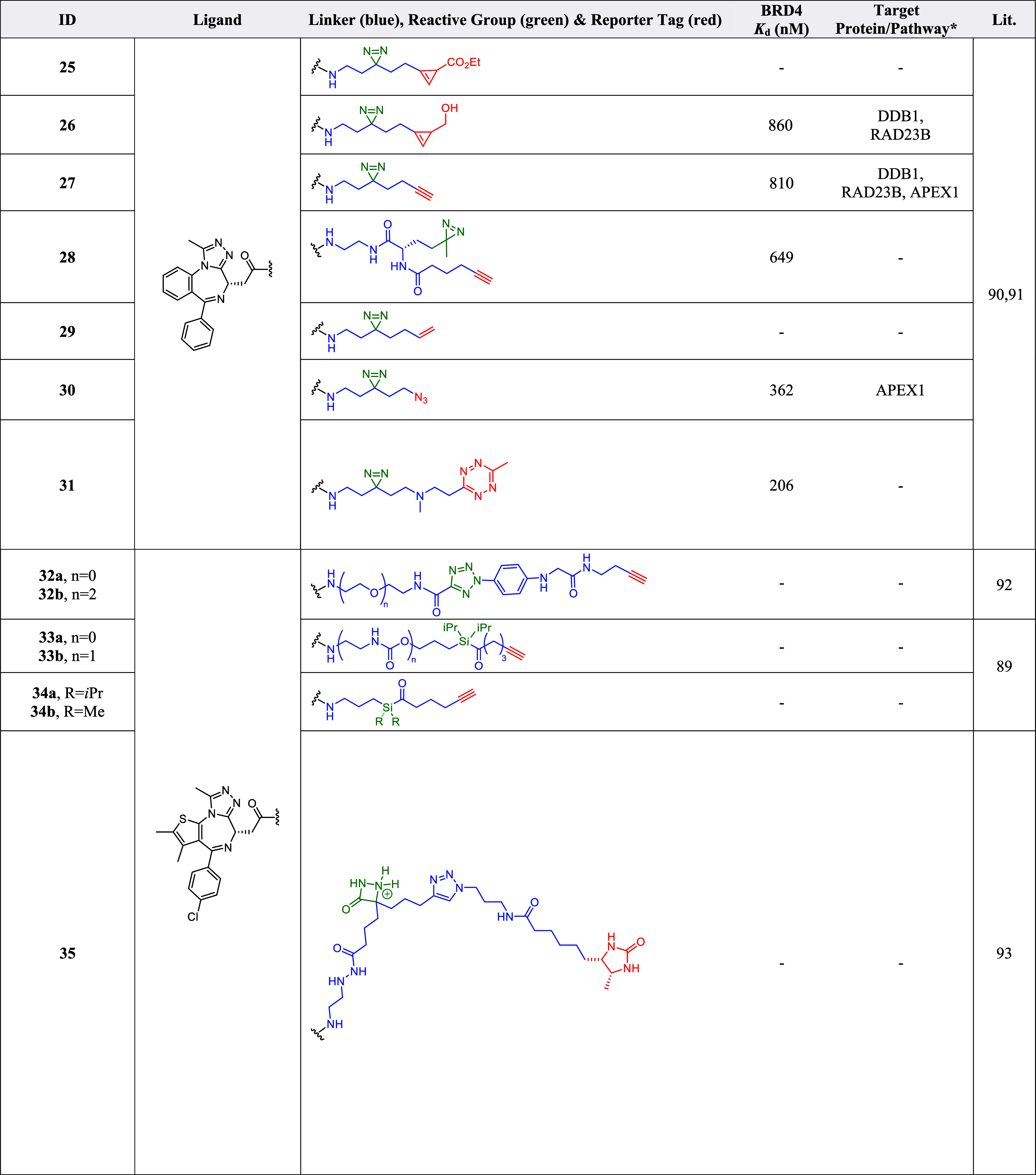

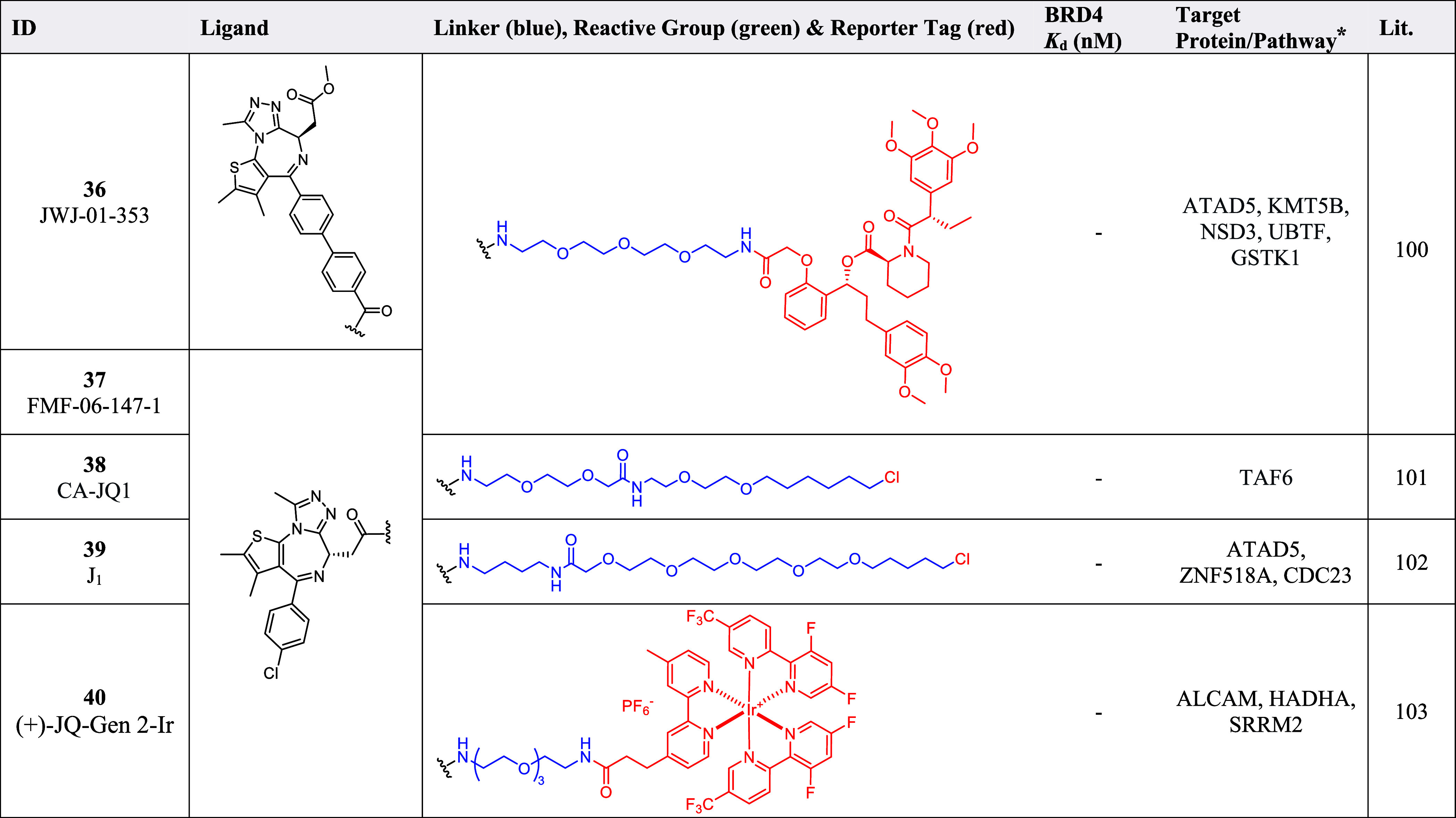
List of Different JQ1-Derivatized Covalent Labeling Probes^a^

a The blue portion represents the linker while the red portion represents reporter tag (e.g. reactive group) within the probes. ALCAM = Activated leukocyte cell adhesion molecule, APEX1 = Apurinic/Apyrimidinic endodeoxyribonuclease 1, ATAD5 = ATPase family AAA domain containing 5, CDC23 = Cell division cycle 23 homologue, DDB1 = DNA damage-binding protein 1, DSC1 = Desmocollin-1, GSTK1 = Glutathione S-transferase kappa 1, HADHA = Hydroxyacyl-CoA dehydrogenase trifunctional multienzyme complex subunit alpha, KMT5B = Lysine methyltransferase 5B, NSD3 = Nuclear receptor binding SET domain protein 3, RAD23B = UV Excision repair protein RAD23 Homologue B, SRRM2 = Serine/Arginine repetitive matrix 2, UBTF = Upstream binding transcription factor, TAF6 = TATA-box binding protein associated factor 6, UBTF = Upstream binding transcription factor, ZNF518A = Zinc Finger Protein 518A. * Proteins enriched besides BET proteins.

Aryl-5-carboxytetrazole (ACT) was demonstrated to be an efficient photoaffinity label for target capture and identification.^92^ ACT provided a unique mechanism of target capture through the photogenerated carboxy-nitrile imine intermediate, which is able to form a covalent bond with a proximal nucleophile near the target active site.^92^ Probe **32** (Table 3), a (+)-JQ1 (**4**) derived analog containing an ACT photoaffinity label, was compared to analogous probes with diazirine and benzophenone moieties.^92^ Among the photoaffinity labels tested, ACT led to the cleanest and most efficient cross-linking when using recombinant target proteins *in vitro*.^92^

A study on a new class of photoaffinity labeling warheads based on UV-activated 1,2-photo-Brook rearrangement of acyl silanes revealed that JQ1-derived *i*Pr-substituted acyl silanes **33** and **34** (Table 3) exhibited superior labeling with minimal background compared to other substituted acyl silanes.^89^ Probes **33** and **34** demonstrated similar level of labeling efficiency with minimal background as analogous diazirine probes in cellular lysate using recombinant BRD4­(BD1).^89^ Though initial attempts to identify protein adducts by mass spectrometry approaches were not successful, acyl silanes were shown to allow modular substitution on the photoactive moiety, offering the potential for direct tuning of desired steric and electronic properties of the probe.^89^

### Electroaffinity Labeling

Electroaffinity labeling provides a new avenue for covalently labeling target proteins via electrochemical activation with a small, redox-active diazetidinone (DZE) functional group to enable chemoproteomic-based target identification of pharmacophores within live cell environments.^93^ This approach avoided the usage of high-energy UV light photoactivation which can complicate downstream target identification and expanded the methodologies applicable for controlled activation of chemical probes to covalently label their protein target in chemoproteomics studies.^93^ A comparison was conducted between electrolysis in DZE probe **35** and UV irradiation on analogous diazirine photochemical probes, revealing a broader amino acid labeling coverage achieved by the DZE probes.^93^ The residue labeling patterns observed, when using DZE, showed covalent binding with aspartate, glutamate, lysine and tyrosine residues, while diazirine group showed glutamate and aspartate residues as the major amino acids labeled.^93^

## Proximity Labeling

Proximity labeling (PL) has rapidly emerged as a robust technique to profile biomolecular interactions with high spatial and temporal precision.^99^ It involves tagging the endogenous interacting partners of target protein (bait) to promiscuous enzymes that catalyze the generation of reactive species in living cells.^99^ Tagged biomolecules/interactomes interacting with target bait protein can then be identified by further analysis with mass spectrometry or nucleic acid sequencing.^99^ Compared to photoaffinity probes, these catalysts label with higher spatial and temporal precision upon blue or red light activation respectively, to identify interactomes of target proteins.^99^

### Biotin Targeting Chimera (BioTAC)

JWJ-01-353 (**36**) and FMF-06-147-1 (**37**) (Table 3) comprising of (+)-JQ1 (**4**) tethered to orthoAP1687 via a PEG linker, a selective binding ligand for the single point mutant of FKBP12^F36V^, were synthesized for validation of the novel biotin targeting chimera (BioTAC) proximity-labeling system.^100^ Both compounds were distinctly different in their structure, with the linker attachment of **36** at the chloro atom of (+)-JQ1 (**4**), while **37** was linked via the commonly utilized carbonyl group.^100^ The BioTAC system utilized a universal recruitable biotin ligase chimera (miniTurboFKBP12^F36V^) expression construct to enable application to any system of interest without the need for cloning or cell line modification.^100^ Successful enrichment of several interactomes were identified with both **36** and **37**, including BRD2, BRD3, BRD4, and a number of BD interactors such as ATAD5, lysine methyltransferase 5B, nuclear receptor binding SET domain protein 3 and upstream binding transcription factor.^100^ Numerous chromatin-associated proteins (Table 3) were also identified by **36**, but not **37**, and vice versa, indicating that different linker conjugation sites allow access to, and labeling of, different ligand-bound target complexes.^100^

### Drug-ID/Ligand-Mediated Proximity Labeling

Drug-ID is another novel method that applies proximity biotinylation to identify drug–protein interactions inside living cells.^101^ The covalent conjugation of a drug with a biotin ligase enables targeted biotinylation and identification of the drug-bound proteome.^101^ CA-JQ1 (**38**) comprising (+)-JQ1 (**4**) and 1-chloroalkane, tethered via a PEG linker, was synthesized for the covalent attachment of (+)-JQ1 (**4**) to the biotin ligase.^101^ With this approach, TATA-box binding protein associated factor 6 was found to be enriched, together with BET family members BRD2 and BRD4, indicating successful enrichment and analysis of (+)-JQ1 interacting protein targets with this approach.^101^ A similar approach was demonstrated by another group with chloroalkane **39** by using cell functionalized with HaloTag proteins to find protein targets and complex members.^102^

### Photocatalytic Proximity Labeling

Photocatalytic proximity labeling in particular has gained significant interest in recent years due to the high level of spatial and temporal control enabled by light activation.^95^ MicroMap (μMap), a method based on a novel iridium-based photocatalyst, was shown to be superior to PAL probes due to the capability to catalytically label more distal residues on the target protein and map the ligand binding site.^95^ μMap-Red^94^ and μMap^95^ are two photocatalysts that have been developed to label target protein catalytically in proximity labeling experiments.^94,95^ Additionally, when used in a cellular context, μMap also allows tagging of other proteins than the target protein that are in close proximity, for instance permitting the identification of multiprotein complexes.^95,103^ The iridium-based derivative (+)-JQ-Gen 2-Ir (**40**) (Table 3) and its inactive enantiomers was able to label residues H77, Q78, F79, A80, W81, and Q84, which are all within 15 Å of the known (+)-JQ-1 (**4**) binding site.^103^ The cell-permeable probe **40** was also successfully used in live Jurkat cells and showed enrichment of BRD4 and other BD reader proteins besides the identification of three covalently modified residues of BRD4, which were located less than 10 Å from the (+)-JQ-1 (**4**) ligand pocket.^103^ These experiments were crucial in demonstrating the powerfulness of μMap for binding site mapping of drugs in close proximity with high specificity in complex native proteomes.^103^ More recently, this technology has been further advanced by the development of a red-light-excited Sn^IV^ chlorin e6 catalyst that is able to activate a phenyl azide biotin probe (μMap-Red).^94,103^ This catalyst activates at a higher wavelength of 660 nm and above, exhibiting superior light-based proximity labeling in complex tissue environments and animal models compared to μMap.^94,103^

The availability of a wider variety of reactive motifs will support target elucidation, both by providing additional tools for the capture of protein targets as well as alternative synthetic approaches for a given probe of interest.^89^ Ideally, the novel reactive motifs cause minimal cellular and protein damage during activation, rapidly generating short-lived reactive intermediate capable of residue-agnostic cross-linking, and minimally perturbing the native substrate–protein interactions.^89^

## Protein Degraders

Protein degraders, such as molecular glue degraders and bifunctional PROTACs, work by recruiting E3 ligases in close proximity to the protein target, inducing ubiquitination followed by degradation by the proteasome system.^53,104^ Molecular glues are small molecules that can change the protein–protein interactions and interactomes by degrading, stabilizing, or activating the target protein after binding,^105^ while PROTACs, are bifunctional molecules that consist of a protein target ligand, linked to an E3 ligase recruiter/inhibitor.

Degraders have the ability to degrade protein targets and assimilate the function of genetic “knockout”, thus serving as valuable tools to examine protein functions.^54^ Additionally, degraders permit the study of target engagement via dose-dependent degradation of the target protein in relation to the respective protein function.^53,54^ Due to the absence of or the need for tight binding with the target protein to initiate protein degradation, degraders are able to target protein–protein interactions with large surface area in conventionally “undruggable” proteins.^53^ Targeted degraders such as molecular glues and PROTACs are currently widely utilized as chemical probes.^53,54^ In recent years, the variation of degrader tool compounds has greatly increased (Figure 4), autophagy-targeting chimeras (AUTACs) that utilizes autophagy-based protein degradation; bacterial PROTACs (BacPROTACs) leveraging degradation pathways in bacteria; photocaged PROTACs (*pc*PROTACs) that allow the timely release of PROTACs using light activation; photochemically targeting chimeras (PHOTACs) that can be activated and inactivated by light; click released PROTACs (*cr*PROTACs) that utilized click chemistry to release and activate PROTACs masked with bioorthogonal clickable groups; macrocyclic PROTACs that utilized macrocyclization to enhance the potency of PROTACs; specific and nongenetic inhibitor of apoptosis protein-dependent protein erasers (SNIPERs) that degrade both the ligand and inhibitor of apoptosis protein; in-cell click-formed proteolysis targeting chimeras (CLIPTACs) that form a PROTACs within the cellular system utilizing click chemistry; and trivalent PROTACs that have the potential of targeting multiple targets or have multiple functionalities. Other degraders such as antibody PROTAC (Ab-PROTACs), folate-PROTACs and enzyme-activating PROTACs utilizing the specificity of antibodies, receptors and enzymes respectively, allow for targeted delivery to cancer cells, overcoming toxicity issues and side effects caused by degradation of target proteins in noncancerous cells. These novel concepts and inventions seek to offer potential pathways to overcome toxicity issues faced by some PROTACs in clinical trial.

**Figure 4:**
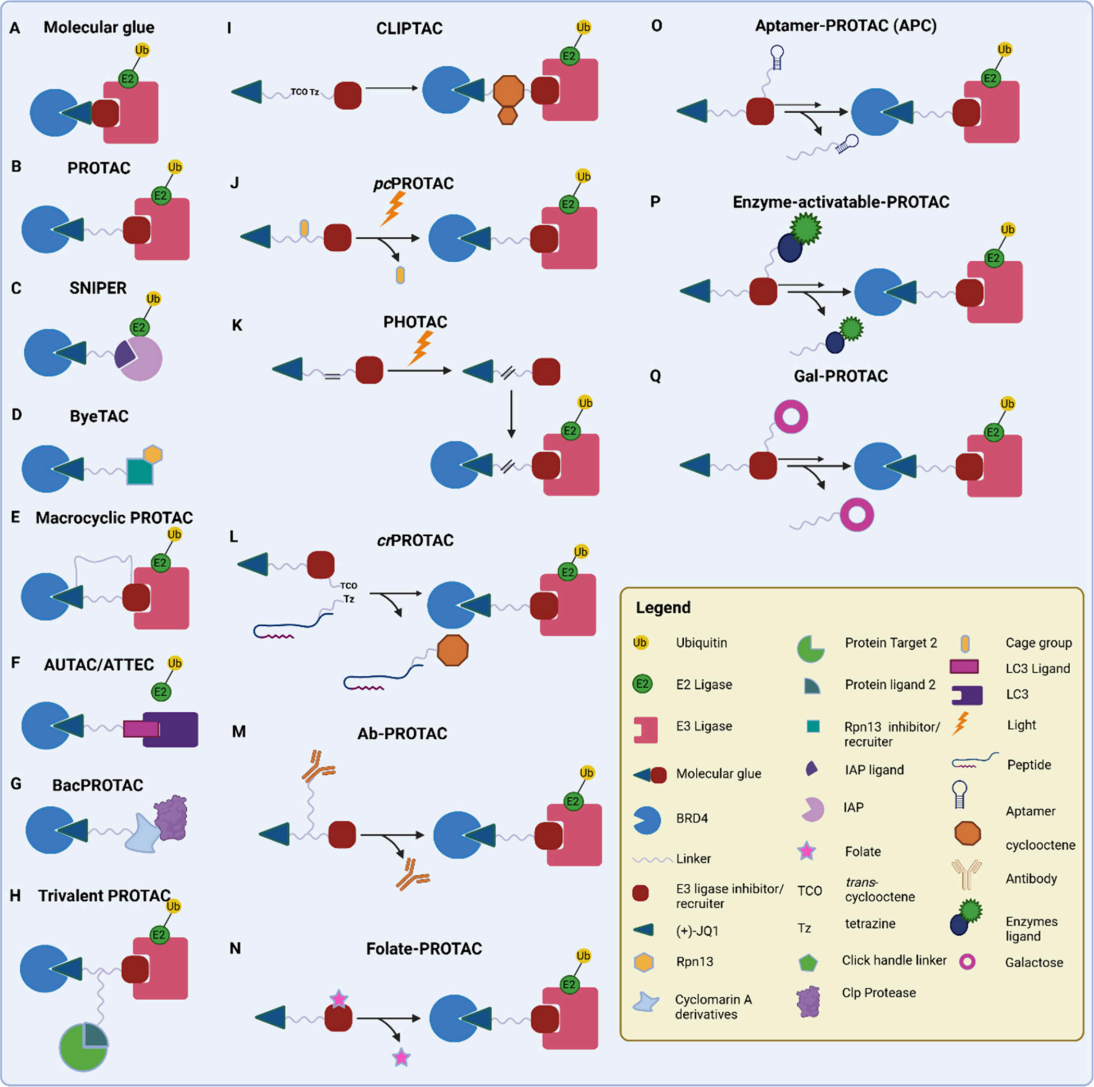
Schematic representation of the different types of JQ1 degraders. (A) Molecular glues, (B) PROTACs, (C) SNIPERs, (D) ByeTACs, (E) Macromolecular PROTACs, (F) AUTAC/ATTECs, (G) BacPROTACs, (H) Trivalent PROTACs, (I) CLIPTACs, (J) Photocaged PROTACs, (K) PHOTACs, (L) Click-release PROTACs, (M) Antibody-PROTACs (Ab-PROTACs), (N) Folate-PROTACs, (O) Aptamer-PROTACs, (P) enzyme-activatable PROTACs, and (Q) Gal-PROTACs. Created in BioRender. Jörg, M. (2025) https://BioRender.com/d84o801.

With the growing interest in degrader probes, Workman and co-workers have also proposed quality criteria for degrader probes, which were recommended to have a *D*_max_ of 80%, and a DC_50_ < 1 μM with at least a 10-fold margin of safety regarding general cytotoxicity in the same cell line.^54^ MS-based proteomics was also mentioned as a quantitative method to assess the degradation of endogenous proteins.^54^ It is noteworthy that PROTACs exhibit a “hook effect”, where the formation of a protein target-PROTAC or E3 ligase-PROTAC binary complex compete with ternary complex formation, impacting the efficacy of PROTACs.^105,106^ Several reviews have been published on the therapeutic effects of BET protein degraders,^57,107–109^ whereas in this review, we will discuss key (+)-JQ1 (**4**) derivatized degraders tools that have led to significant breakthroughs in novel applications and discoveries.

### Molecular Glues

(+)-JQ1-derived covalent molecular glues GNE11 (**41**), TMX1 (**42**), MMH1 (**43**), and MMH2 (**44**) (Table 4) were developed by modifying the chloro-substituent of (+)-JQ1 (**4**) and installing DNA damage-binding protein 1 (DDB1)- and Cullin-4A (CUL4)-associated factor 16 (DCAF16) recruiting moieties propargylic amine, enone, acrylamide and vinyl sulfonamide, respectively.^110^ The electrophilic nature of the introduced Michael acceptors resulted in the formation of covalent bonds with E3 ligase DCAF16.^110^ These molecular glues were found to bind specifically to BRD4­(BD2) over BRD4­(BD1), and displayed improved degradation potency over analogs without the electrophilic warheads.^110^ Degradation potency of these compounds correlated to the reactivity of the covalent warhead, with acrylamide and vinyl sulfonamide groups containing molecular glues, **43** and **44**, respectively, displaying better degradation potency than GNE11 (**41**) and TMZ1 (**42**).^110^ Immunoprecipitation mass spectrometry (IP-MS) experiments and cryogenic electron microscopy studies found the covalent modification on DCAF16 to be facilitated through the binding with BRD4­(BD2) complex, instead of direct reactivity with DCAF16 alone.^110^ IP-MS studies also revealed cyclin G-associated kinase and endoplasmic reticulum resident protein 29 to bind to BRD4­(BD2) in the presence of covalent analogs **42**–**44**, which was absent in noncovalent analogs.^110^ This work successfully demonstrated the feat of (1) utilizing a strong covalent E3 ligase recruiter to improve degradation potency, (2) degradation of (+)-JQ1-derived molecular glues recruiting DCAF16, and last (3) installing electrophilic warheads at the *para*-position of the phenyl ring of (+)-JQ1 (**4**) molecule for the development of (+)-JQ1-derived molecular glues.^110^

**Table 4:**
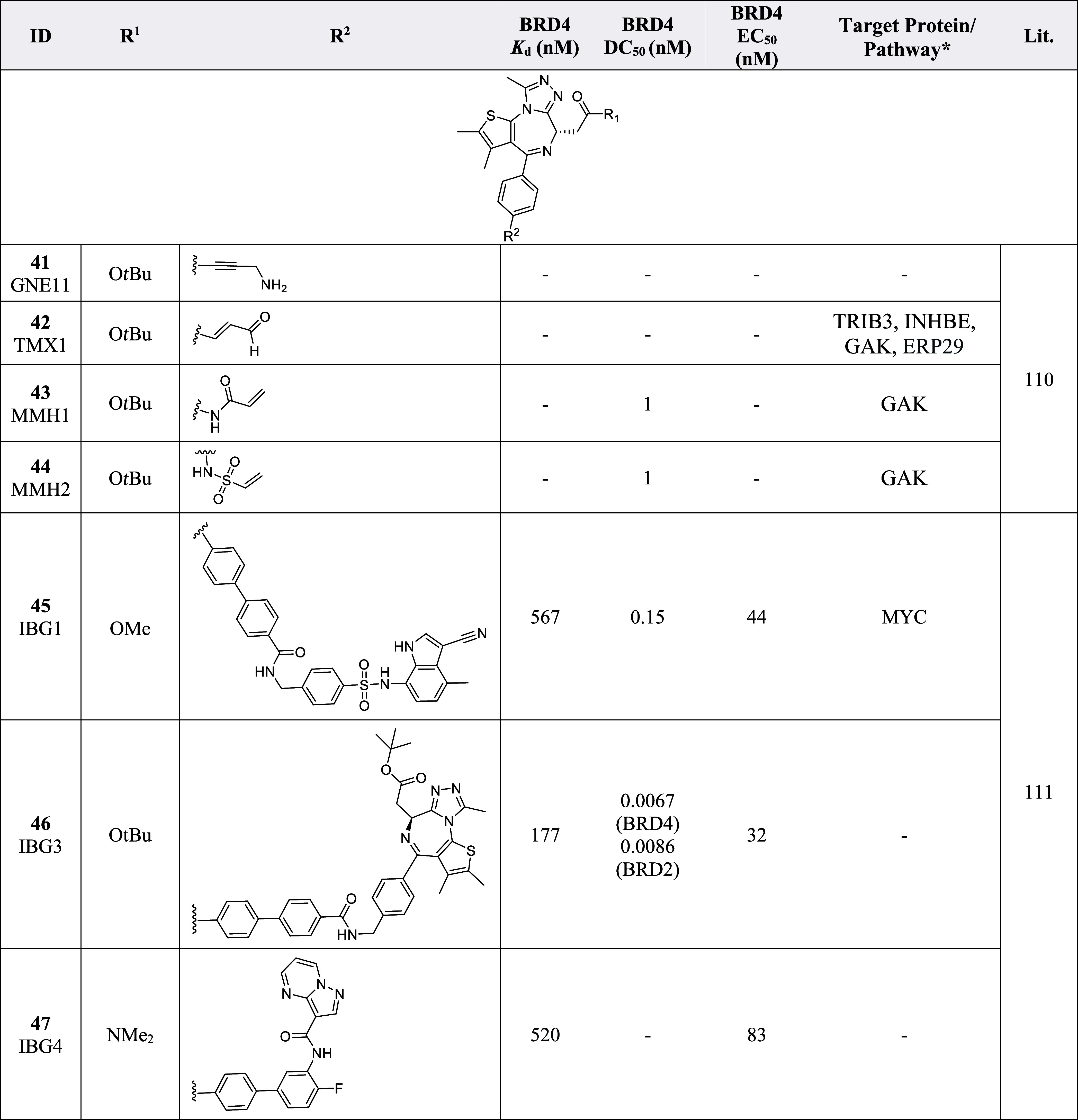
List of Different JQ1-Derivatized Molecular Glue Probes with Different Substituents^a^

a ERP29 = Endoplasmic reticulum resident protein 29, GAK = Cyclin G-associated kinase, INHBE = Inhibin beta E chain, TRIB3 = Tribbles homologue 3. *Non-BET enriched proteins.

An alternative strategy to traditional PROTACs or molecular glues termed intramolecular bivalent glues (IBGs) has been developed to potently and selectively degrade BRD2 and BRD4.^111^ IBG1 (**45**), IBG3 (**46**) and IBG4 (**47**) (Table 4) have shown to concurrently bind to two distinct sites on the protein target to initiate the formation of stable ternary complexes, leading to the degradation of the target protein.^111^ IBG1–3 (**45**–**47**) showed excellent degradation potencies, with IBG3 (**46**) exhibiting low picomolar activity with a DC_50_ of 6.7 pM and 8.6 pM, respectively.^111^ For IBGs **45** and **47**, the degradation was induced via DCAF11 or DCAF16 ligands, respectively.^111^ While this is the first report of such a mechanism of action for these IBGs, it remains to be seen if the approach is applicable to other protein families.

### Proteolysis Targeting Chimeras (PROTACs)

Despite the discovery of PROTACs in 2001 by Crew’s and co-workers,^112^ the first small molecule PROTACs were only disclosed in 2015. The development of MZ1 (**48**), dBET1 (**50**), and ARV-825 (**56**) (Table 5) was signifying an important breakthrough in PROTAC research.

**Table 5:**
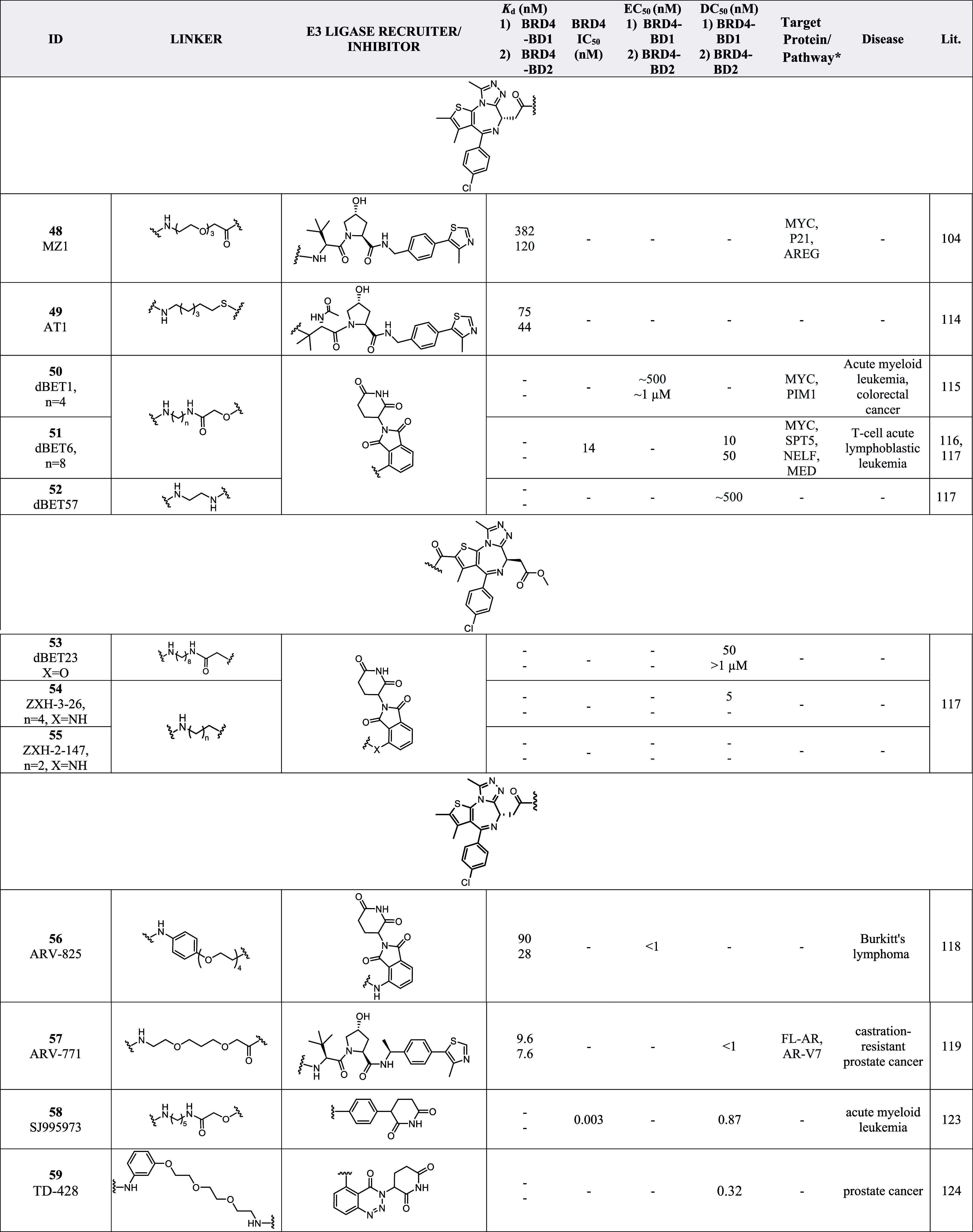

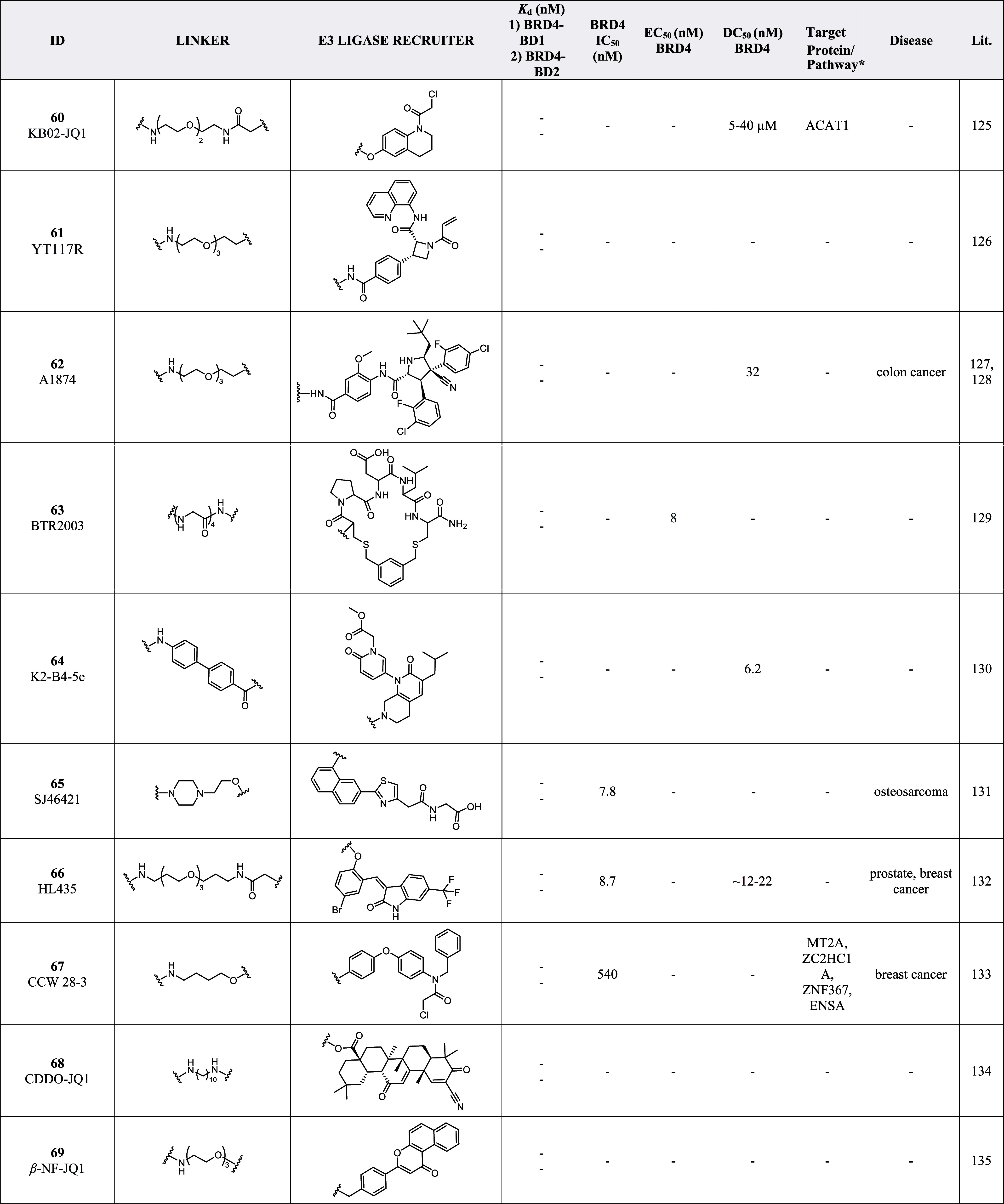

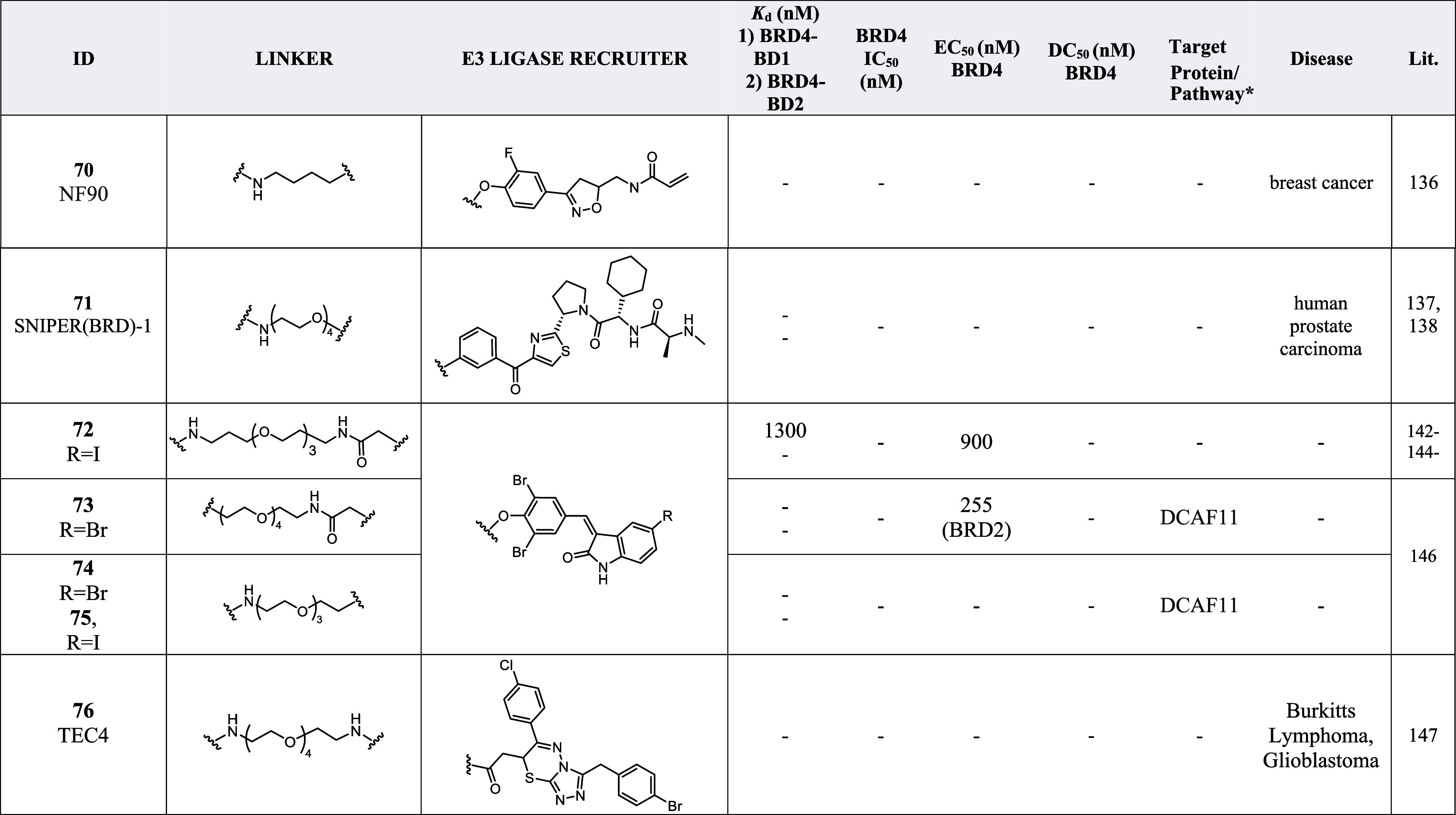
List of JQ1-Derivatized PROTACs with Different E3 Ligase Recruiter/Inhibitors and Linkers and Their Biological Activities^a^

a ACAT1 = Acetyl-CoA acetyltransferase 1, AREG = Amphiregulin, AR-V7 = Androgen receptor variant 7, DCAF11 = DDB1 and CUL4 associated factor 11, ENSA = Endosulfine alpha, ARFl = AR full length, MYC = Myelocytomatosis, MT2A = Metallothionein 2A, NELF = Negative elongation factor, P21 = Cyclin-dependent kinase inhibitor 1A, PIM1 = Serine/threonine-protein kinase, SPT5 = Transcription elongation factor, ZC2HC1A = Zinc finger C2HC domain-containing protein 1A, ZNF367 = Zinc finger protein 367. *Non-BET enriched proteins.

PROTAC **48** consists of (+)-JQ1 and VHL032 (a von Hippel–Lindau (VHL) E3 ligase recruiter) connected by a PEG linker.^104^ In this work, a series of PROTACs with different linker lengths were tested, and **48** was found to display comparable binding affinities to that of (+)-JQ1 (**4**) for BRD4­(BD1) and BRD4­(BD2) of the BET family protein.^104^ In comparison to other PROTACs with longer linker lengths, **48** was found to display the best degradation potency, and showed preferential degradation of BRD4 over BRD2 and BRD3 proteins,^104^ suggesting the importance of the linker length in PROTAC design and their effect on polyubiquitination initiation due to proximity between the ligand and E3 ligase recruiter.^113^ On the other hand, negative control *cis*MZ1, with a different stereochemistry at the hydroxyl moiety of VHL032, did not show any binding affinity for VHL in the isometry titration calorimetry experiment, emphasizing the importance of the E3 ligase activity in protein degradation initiation.^104^ PROTAC **48** also demonstrated selective depletion of BRD4 compared to inhibiting the whole BET protein subfamily with (+)-JQ1 (**4**), suggesting a different pharmacological response.^104^ This work successfully demonstrated the possibility of utilizing PROTACs as new tools for studying BD and their validation.^104^ The first crystal structure of **48** bound to both E3 ligase and target protein (#5T35), showed **48** to be “sandwiched” between the two proteins.^114^ Biophysical binding studies determined that ternary complexes formation was influenced by the specificity of cooperative recognition caused by induced protein–protein interaction contacts.^114^ Guided by the crystal structure obtained, AT1 (**49**) (Table 5), with a shorter six-carbon chain linker and an acetyl group on the VHL-like recruiter was developed.^114^ **49** showed exquisite selectivity in the depletion of BRD4 over BRD2 and BRD3 and was revealed to form a greater cooperative ternary complex with BRD4­(BD2).^114^

The celebron (CRBN)-targeting PROTAC, dBET1 (**50**) (Table 5) links (+)-JQ1­(**4**) to thalidomide (a CRBN E3 ligase recruiter) via a four-carbon alkyl chain.^115^ Chemical genetic and gene-editing approaches were applied to explore the mechanism of **50**-induced BRD4 degradation, which confirmed that treatment with either (+)-JQ1 (**4**) or thalidomide alone were unable to induce BRD4 degradation, and that the proteasomal degradation of BRD4 is CRBN dependent.^115^ This concept of utilizing degrader as a chemical biology tool demonstrates the feasibility of approaching intractable protein targets using phthalimide-conjugation of target-binding ligands in a broader application.^115^ PROTAC **50** was further optimized to give dBET6 (**51**) (Table 5), which comprised of a longer eight-carbon linker chain, that displayed improved efficacy and selectivity for T-cell acute lymphoblastic leukemia *in vitro* and *in vivo*.^116^ The improved membrane permeability of **51** was confirmed via standardized colorectal adenocarcinoma (Caco)-transwell assays, explaining its higher cellular potency (IC_50_ = 14 nM), compared to **50** and other similar PROTACs with shorter alkyl linkers.^116^ It was also found that the initiation of BRD4 degradation was correlated with cellular toxicity, prompting subsequent downregulation of MYC and induction of apoptosis.^116^ With probe **51**, it was discovered that BET degradation led to chromatin displacement of different regulatory factors and established BD (BRD4-BD in particular) as master regulators of global transcription elongation through the assemble of a productive transcription elongation complex.^116^ It was also demonstrated in this work that BET degradation was mechanistically different from BD inhibition due to its capability to interfere with this essential gene regulatory mechanism.^116^

With PROTACs dBET57 (**52**), dBET23 (**53**), ZXH-3-26 (**54**), and ZXH-2-147 (**55**) (Table 5), the molecular basis of degrader-mediated neo-substrate recruitment to cullin4A-RING E3 ubiquitin ligase/CRBN (CRL4^CRBN^) ligase complex that comprised of CRBN, DDB1, ring-box 1, and the scaffold protein CUL4A was determined via a combination of structural, biochemical and cellular data.^117^ Different from most of the (+)-JQ1 (**4**)-derived PROTACs, **53** is connected to the thiophene group of (+)-JQ1 (**4**), instead of the common carbonyl attachment point. Compound **53** contains an eight-carbon linker attached to thalidomide via an oxyacetamide linkage, while **52** comprises of a shorter ethylenediamine linker attached to the common carbonyl attachment site of (+)-JQ1 (**4**).^117^ Crystal structures, showed that the different selectivity profiles of **52** and **53** might be explained by interactions with different surface residues of the protein target, further highlighting the impact of linker length and attachment location in the design of PROTACs.^117^ It was demonstrated that highly effective and selective degraders can be attained despite weak binding affinity in interprotein contacts and the lack of tight binding or positive cooperativity.^117^ Similarly, it was revealed binding between the ligase and the substrate was flexible, and distinct conformations were adapted depending on linker length and position.^117^ Hence, the development of degraders can be guided using *in silico* protein docking exploiting “local” energy/entropy minima to achieve selectivity.^117^ A cooperativity factor alpha (α_app_ = IC_50_[binary]/IC_50_[ternary]) was defined with binding experiments in the presence of increasing concentrations of either BRD4­(BD1) or BRD4­(BD2), to assess the cooperativity of ternary complex formation.^117^ Positive cooperativity resulted in α_app_ > 1 while negative cooperativity in α_app_ < 1.^117^ Compared to PROTAC **53**, **54** was reported to display selective targeting of BRD4 over BRD2 and BRD3.^117^ This denotes the feasibility of achieving pharmacological selectivity through distinct conformation that induced protein–protein interactions despite the distance from the binding site of the molecule.^117^

CRBN-targeting PROTAC ARV-825 (**56**) (Table 5) has an aromatic ring as part of its PEG linker chain, and was able to achieve a high degradation potency (DC_50_) of below 1 nM for BRD4, despite its low binding affinity.^118^ This discovery opened up opportunities to develop functional degraders, consisting of target ligands with insufficient affinity to provide a functional effect on targets with shallow pockets, which may become druggable with PROTAC-mediated degradation.^118^ VHL-targeting PROTAC ARV-771 (**57**) (Table 5) demonstrated high activity against cellular models of castration-resistant prostate cancer (CRPC) with rapid BET protein degradation of DC_50_ < 1 nM and degraded BRD2/3/4 in human prostate carcinoma epithelial cell line 22Rv1 cells with a DC_50_ < 5 nM.^119^ Treatment with **57** was also found to result in the depletion of MYC, a downstream effector of BET proteins, with an IC_50_ < 1 nM.^119^ Subcutaneous delivery of **57** in two different mouse models of CRPC resulted in tumor regression in enzalutamide-resistant 22Rv1 xenografts, validating BET protein degradation as a promising clinical strategy against metastatic CRPC and demonstrating the feasibility of treating solid-tumor malignancies with PROTACs.^119^

ARV-825 (**56**) and ARV-771 (**57**) are both currently in clinical trials, while MZ1 (**48**), dBET1 (**50**) and dBET6 (**51**) are often utilized in many other works to probe the biological system of BRD4. With **48** and **50**, it was found that the amount of the respective E3 ligases present in the cell was the determining factor for the efficiency of degradation, which can be employed to develop resistance cells by downregulating the level of the E3 ligase.^120^ VHL-based PROTACs were found to entail broader utility in cancer cell lines compared to CRBN-targeting PROTACs, as CRBN protein levels were found to be frequently suppressed in cancer cell lines.^121^ It was also revealed that the linker utilized in a PROTAC molecule mostly impacted the metabolic stability of PROTACs, with the attachment points to the ligands being most labile.^122^ Specifically, human cytochrome P450 3A4 was recognized to be involved in *N*-dealkylation and amide hydrolysis of PROTACs, and human aldehyde oxidase was found to have a crucial role in the metabolism of VHL PROTACs.^122^ This discovery is noteworthy to medicinal chemists in their pursuit of more metabolically stable PROTACs.

Phenyl-glutarimides were reported as a more stable substitute for inherently unstable thalidomide and structurally related immunomodulatory imide drugs (IMiDs), such as pomalidomide and lenalidomide, which readily undergo hydrolysis in body fluid, media and buffer.^123^ The phenyl-glutarimides (+)-JQ1-PROTACs were compared with analogous thalidomide (+)-JQ1-PROTACs, revealing improved stability and degradation potency.^123^ Among them SJ995973 (**58**) (Table 5) was found to display one of the highest cellular potencies in reported BET PROTACs with an IC_50_ value of 3 pM, and BRD4 DC_50_ = 0.87 nM (*D*_max_ = 99%) in MV4-11 cells, demonstrating the phenyl-glutarimides to overcome the hydrolysis tendency of (IMiDs) functionalities.^123^

Aminobenzotriazino glutarimide TD-106 was revealed as a novel IMiD analog superior to pomalidomide.^124^ The degradation of Ikaros zinc finger like IMiDs by TD-106 was found to be comparable to pomalidomide in immunoblotting analysis.^124^ When TD-106 was linked to (+)-JQ1 (**4**) to afford PROTAC TD-428 (**59**) (Table 5), BRD4 degradation in 22Rv1 cell was induced with a DC_50_ of 0.32 nM, a better degradation potency than ARV-825 (DC_50_ = 0.57 nM).^124^ The superior degradation potency of **59** compared to ARV-825, was contributed to TD-106 highlighting its potential as a novel CRBN modulator for targeted protein degradation.^124^

E3 ligase ligands/recruiters are an important component of PROTACs and many works were conducted to expand the E3 ligase toolbox beyond the commonly utilized CRBN and VHL for PROTACs development.^125^ A number of other novel E3 ligase ligands/recruiters were discovered using (+)-JQ1 (**4**) as the target binder to validate novel E3 ligase ligands for future PROTAC research. Containing a cysteine-directed fragment electrophile, electrophilic covalent-binding KB02-JQ1 (**60**) (Table 5) was found to be advantages by inducing degradation with modest binding (40%) to DCAF16, resulting in substoichiometric modification of DCAF16.^125^ Though high concentration (20 μM) of **60** was required to induce detectable degradation, it was able to selectively degrade BRD4 over BRD2 and BRD3 and decrease the level of acetyl CoA acetyltransferase 1 in the proteome.^125^ This work successfully implemented the use of electrophilic warhead to covalently target DCAF16 and expanded the scope of E3 ligases available for future PROTAC development.^125^

Stereo- and site-specific azetidine acrylamide was uncovered to react with cysteine (C1113) in DCAF1 E3 ligase substrate receptor in cysteine-directed mass spectrometry activity-based protein profiling (ABPP) proteomic analysis of stereochemically defined electrophilic compound sets.^126^ Electrophilic PROTAC YT117R (**61**) (Table 5) consisting of (+)-JQ1 (**4**) and the DCAF1-directed azetidine acrylamide in *R*-stereochemistry demonstrated degradation of BRD4 at 0.5 μM in immortalized human embryonic kidney cells HEK293T.^126^ Gel-ABPP experiments confirmed the concentration-dependent and stereoselective blockade of labeling of recombinant DCAF1 by **61**, while the azetidine acrylamide analog with *S*-stereochemistry showed no changes, revealing the first electrophilic PROTAC that has been shown to act in a stereo- and site-selective manner.^126^

The first mouse double minute 2 (MDM2)-targeting BRD4 PROTAC, A1874 (**62**) (Table 5), was developed by attaching nutlin to (+)-JQ1 (**4**) via a PEG linker.^127^ PROTAC **62** was shown to synergistically stabilize p53 and degrade BRD4, displaying superior antiproliferative activity on cancer cell lines when compared to analogous VHL PROTACs.^127^ In contrast to many PROTACs reported, no “hook effect” was observed at up to 10 mM, suggesting that the recruitment of MDM2 was superior compared to recruitment of CRBN and VHL.^127^ A mechanistic study on the therapeutics effect of **62** on colon cancer cells was conducted to elucidate the superior activity of **62** against colon cancer cells due to both BRD4-dependent and BRD4-independent (p53 stabilization and reactive oxygen species production) mechanisms, demonstrating the potential benefits of targeting the E3 ligase MDM2 over CRBN and VHL E3 ligases, which initiate only BRD4-dependent degradation.^128^

Synthetic macrocyclic Kelch-like protein 20 **(**KLHL20) E3 ligase ligand, BTR2000, was developed to confer selectivity on the (+)-JQ1 (**4**) molecule, leading to selectivity of BRD2 and BRD3 over BRD4 protein.^129^ PROTAC molecule BTR2003 (**63**) (Table 5) was developed by linking BTR2000 to (+)-JQ1 (**4**) with a linker consisting of four glycine amino acids, which displayed better affinity than analogous PROTACs with PEG linkers.^129^ Ligand **63** was found to bind to the Cullin 3-Kelch-like protein 20 (CUL3^KLHL20^) complex, allowing the expansion of the E3 ligase beyond the commonly engaged Cullin-4-CRBN (CUL4^CRBN^) and Cullin-2-VHL (CUL2^VHL^) complexes.^129^

PROTAC K2-B4-5e (**64**) (Table 5) has ligand KDRLKZ-1 of Kelch domain-containing protein 2 (KLHDC2) E3 ligase tethered to (+)-JQ1 (**4**).^130^ Compound **64** was shown to have a DC_50_ of 6.2 nM and *D*_max_ of 93% in HiBiT-BRD4 degradation.^130^ Another KLHDC2 ligand SJ46418, was also tethered with (+)-JQ1 (**4**) to afford PROTAC SJ46421 (**65**) (Table 5) with selectivity toward BRD3 in osteosarcoma U2OS cells with DC_50_ of 224 nM and *D*_max_ of 99.9%.^131^ Both works revealed successes in utilizing E3 ligase KLHDC2 that targets C-terminal degrons.

Alkenyl oxindole-based PROTAC HL435 (**66**) (Table 5) was found to recruit E3 ligase complex CRL4^DCAF11^ in the proteasomal degradation of BRD4.^132^ Optimization of the linker length and composition, as well as the alkenyl oxindole E3 ligase recruiters were conducted, leading to the molecule **66**, which displayed the best degradation efficiency with a DC_50_ value of 11.9 nM in breast cancer MDA-MB-231 cells and an IC_50_ of 8.7 nM against 22RV1 cells.^132^

The first ring finger protein 4 (RNF4)-targeting PROTAC, CCW 28–3 (**67**) (Table 5), was found to degrade BRD4 in a RNF4-dependent manner.^133^ The covalent recruiter was discovered via ligand screening, and linked to (+)-JQ1 via a aliphatic alkyl chain to obtain PROTAC **67**, which displayed comparable degradation efficiency to MZ1 (**48**).^133^

PROTAC CDDO-JQ1 (**68**) (Table 5) designed based on the known Kelch-Like ECH- associated protein 1 ligand bardoxolone (CDDO), demonstrated reversible, covalent binding of E3 ligase recruiter CDDO for protein target degradation applications.^134^ Similarly, PROTAC β-NF-JQ1 (**69**) (Table 5) was demonstrated to be an effective PROTAC, attaching (+)-JQ1 (**4**) to β-NF, an aryl hydrocarbon receptor (AhR) ligand able to recruit AhR E3 ligase for the degradation of BET proteins.^135^

Covalent chemoproteomic studies revealed EN67, a covalent recruiter against the E2 ligase UBE2D by targeting an allosteric cysteine C111.^136^ PROTAC NF90 (**70**) (Table 5) consisting of (+)-JQ1 tethered to EN67, successfully demonstrated degradation of BRD4 short isoform with partial degradation from 10 nM to complete degradation at 10 μM in MDA-MB-231 cells.^136^ Interestingly, the compound is selective to the short isoform of BRD4, but not the longer isoform of BRD4, potentially eliminating the oncogenic roles of BRD4 while maintaining its tumor suppressive functions.^136^ This work opened up the potential for recruitment of E2 ligase of the ubiquitin proteasome system (UPS) machinery for targeted protein degradation.^136^ However, further studies would be required to understand if recruitment of the E2 ligase potentially bypasses the necessity for E3 ligase recruitment.^136^

### Specific and Nongenetic Inhibitor of Apoptosis Protein (IAP)-Dependent Protein Erasers (SNIPERs)

SNIPERs, unlike other degraders, do not degrade just the target protein but also induce the simultaneous degradation of inhibitor of apoptosis proteins (IAPs), a class of negative regulators for cell apoptosis.^137,138^ The overexpression of IAPs in cancer cells and their relation to poor prognoses make them important targets for cancer therapy.^139,140^ Belonging to the E3 ubiquitin ligase family IAPs were commonly used as E3 ligase target for SNIPERs, which have been successfully applied in the degradation of different target proteins in various diseases, such as cancer, immune disorders, and neurodegenerative diseases.^138^ The first (+)-JQ1 (**4**)-derived SNIPER degrader, SNIPER­(BRD)-1 (**71**) (Table 5), comprising of IAP antagonist LCL-161 as the IAP ligand, was shown to degrade cellular inhibitor of apoptosis protein-1 (cIAP1), cellular inhibitor of apoptosis protein-2 (cIAP2), and X-linked inhibitor of apoptosis (XIAP) with IC_50_ of 6.8 nM, 17 nM, and 49 nM, respectively, together with BRD4.^137^ The first (+)-JQ1 (**4**)-derivatized SNIPER **71** assisted in the elucidation of the mechanism of degradation with different IAPs, showing binding of the IAP antagonist induced autoubiquitylation of cIAP1, while the SNIPER-induced degradation of XIAP and BRD4 required a ternary complex formation.^137^

### Autophagy-Targeting Chimera (AUTACs)/Autophagosome-Tethering Compound (ATTEC)

AUTACs are heterobifunctional compounds which have protein target ligands linked to a tag, to degrade target proteins via the autophagy system rather than the UPS.^141^ The concept of autophagy-based protein degradation was applied in AUTAC **72** (Table 5) to degrade BRD4 protein by targeting autophagy key protein microtubule-associated proteins 1A/1B light chain 3B (LC3) with a α, β-unsaturated indolinone LC3 warhead, GW5074.^142^ The degradation efficiency of the AUTACs was observed to improve with increased linker length, while the efficiency decreased when the linker was too long, which was congruent with previous studies by other groups.^142^ AUTAC **72** was shown to successfully degrade BRD4 through the autophagy pathway and exhibited good antiproliferative activity in multiple tumor cells.^142^ AUTAC **72** presented a new tool for studying disease-related targets with target specific autophagy-based degradation such as protein aggregates, damaged organelles and lipid droplets.^142^ Compound **72** was also classified as an autophagosome-tethering compound (ATTEC) in a review,^143^ which is a bifunctional molecule composed of three components: a ligand of the protein target, a flexible linker, and an LC3 warhead.^144^ Similar to the AUTACs, ATTECs function by tethering the protein target to the autophagosome, however, AUTAC molecules recruit autophagosomes for degradation, while ATTECs only bind to LC3.^145^

However, later studies with structurally related degraders **73**–**75** (Table 5) differing only in the linkers connecting the LC3 warhead GW5074 with (+)-JQ1 (**4**), revealed induced degradation of their target proteins through the UPS and not via the autophagy pathway.^146^ Subsequent target identification and validation, as well as mechanistic investigations, showed that the 3-arylidene-indolinones in these molecules covalently binds to the substrate receptor protein DCAF11, confirming its mechanism via the UPS.^146^

The development of (+)-JQ1-derivatized AUTACs is shown to be in its infancy stages. It remains a challenge to target the BET proteins located in the nucleus and to link them to the lymphatic system, which requires overcoming distance and barriers between the cellular and nuclear membranes. The above findings and discussions of the aforementioned works signify much room for further exploration and discussions in the work of JQ1-derived AUTACs and ATTECs for targeting BET proteins.

### Bypassing E3 Targeting Chimeras (ByeTACs)

Bypassing E3 targeting chimeras (ByeTACs) utilized a ubiquitin-independent mechanism to overcome the need for an E3 ligase by initiating an interaction between the protein target and Rpn-13, a 26S proteasome subunit.^147^ Being a nonessential ubiquitin receptor for the 26S proteasome in healthy cells, Rpn-13 is required in cells under significant stress or cells requiring ubiquitin-dependent degradation of proteins for survival, such as cancer or inflamed cells.^147^ This study successfully demonstrated the proof of concept that degradation of a protein target can be mediated through a noncovalent interaction with Rpn13.^147^ Bifunctional TEC4 (**76**) (Table 5) consisting of (+)-JQ1 and Rpn-13 ligand (TCL1) was shown to retained its degradation capability in Burkitts Lymphoma Ramos cell when TAK-243, an E1 inhibitor, was added simultaneously to shut down the ubiquitin ligase cascade, confirming that ByeTAC methodology can degrade a protein without the recruitment of ubiquitin.^147^ On the other hand, ARV-825 degradation capability was lowered when the ubiquitin ligase cascade was shut down. Compound **76** displayed discriminative degradation between Ramos and HEK293T cells in cell viability experiments with a 50-fold higher concentration required to degrade BRD4 in noncancerous HEK293T, demonstrating its ability to distinguish between cancerous and healthy cell lines.^147^

### Bacterial PROTACs (BacPROTACs)

The PROTAC technology is so far restricted to the ubiquitin tagging system of eukaryotes and has yet to be transferred to degradation pathways in prokaryotic bacteria. In order to explore the possibility of expanding the targeted protein degradation mechanism to bacteria cells, Clausen et al. applied the BRDT-degron system by expressing BRDT_BD1_ in *Mycobacterium smegmatis* *.* ^148^ As a human protein with no human homologue, utilizing the BRDT_BD1_ degron would prevent interfering with endogenous pathway in the bacteria.^148^ Additionally, in order to enable the ClpC-mediated protein degradation through the involvement of caseinolytic protease proteolytic subunit (ClpC:ClpP (ClpCP)) protein (the equivalent of the eukaryotic proteasome), the ClpC1_NTD_ substrate receptor domain, which is targeted by a known antibiotic, Cyclomarin A (CymA), was selected.^148,149^

Bacterial PROTACs (BacPROTACs) comprising of BRDT-targeting (+)-JQ1­(**4**) and ClpC1-targeting CymA-derived cyclic peptides, sCym-1 and dCymM, both possessing high binding affinity to ClpC1_NTD_, K_D_ = 0.81 μM and 0.2 μM respectively, were linked with PEG or alkyl linker to obtain BacPROTAC-3-5 (**77**–**79**).^148^ The triazole ring was also introduced in the linker in **78** and **79** and was found to pose no hindrance in binding affinity. Capillary Western blot analysis revealed **77**–**79** to exhibit degradation of BRDT and bacteriotoxicity at 10 μM in *M. smegmatis* with the BRDT_BD1_ degron.^148^ Proteomics analysis also confirmed the selectivity of BacPROTACs **77** by identifying BRDT as the only protein dysregulated.^148^

Nonetheless, for the system to work successfully in bacterial cells with antimicrobial potential, endogenous bacterial proteins would have to be utilized.^148^ Through the above approach, they fused d-alanine ligase A (DdlA) and threonine synthase (ThrC) with the BRDT_BD1_-degron and validated them with BacPROTACs as suitable essential bacterial target proteins for future antimicrobials development.^148^ This work successfully demonstrated the feasibility of using BacPROTAC-induced degradation with high selectivity and species specificity by reprogramming the ClpCP protease in bacterial cells and was shown as a proof of concept for development of novel antimicrobial agents utilizing a degradation mechanism.^148^

Further work utilizing BacPROTACs to discover the function of ClpC1/2/3 in *M. tuberculosis* was also reported but this is beyond the context of this review and will not be further discussed.^150^

**5:**
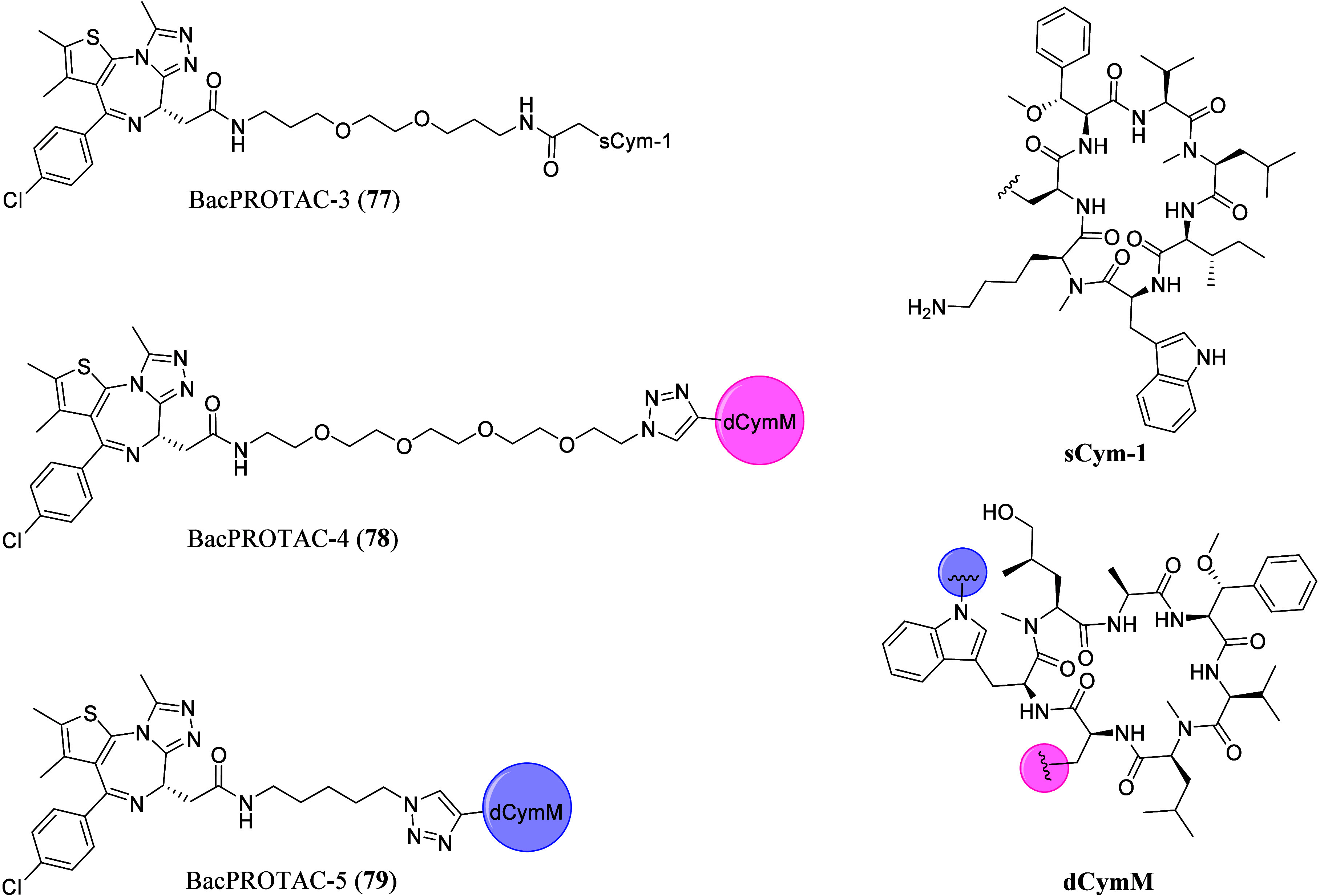
Chemical structures of (+)-JQ1 (**4**) containing BacPROTACs **77**–**79**. Compound **77** is linked to sCym-1, whereas analogs **78** and **79** are linked to dCymM via different linkers and conjugation sites.

### Macrocylic PROTACs

Macrocyclization is an attractive strategy to constrain a molecule in its bioactive conformation.^151^ MacroPROTAC-1 (**80**) (Figure 6) was developed by adding an additional PEG linker to cyclize PROTAC MZ1 (**48**).^151^ X-ray crystallography was used to obtain a cocrystal structure of macroPROTAC-1 bound in a ternary complex with VHL and BRD4­(BD2).^151^ In biophysical studies, **77** showed enhanced selectivity for BD2 over BD1, and exhibited cellular activity comparable to MZ1 (**48**), in HeLa cervical cancer cells and 22RV1 cells, despite a 12-fold loss of binary binding affinity for BRD4.^151^ This work supported macrocyclization as a strategy to enhance PROTAC degradation potency and selectivity between homologous targets despite the bulkier structure and increased molecular size.^151^

**Figure 6:**
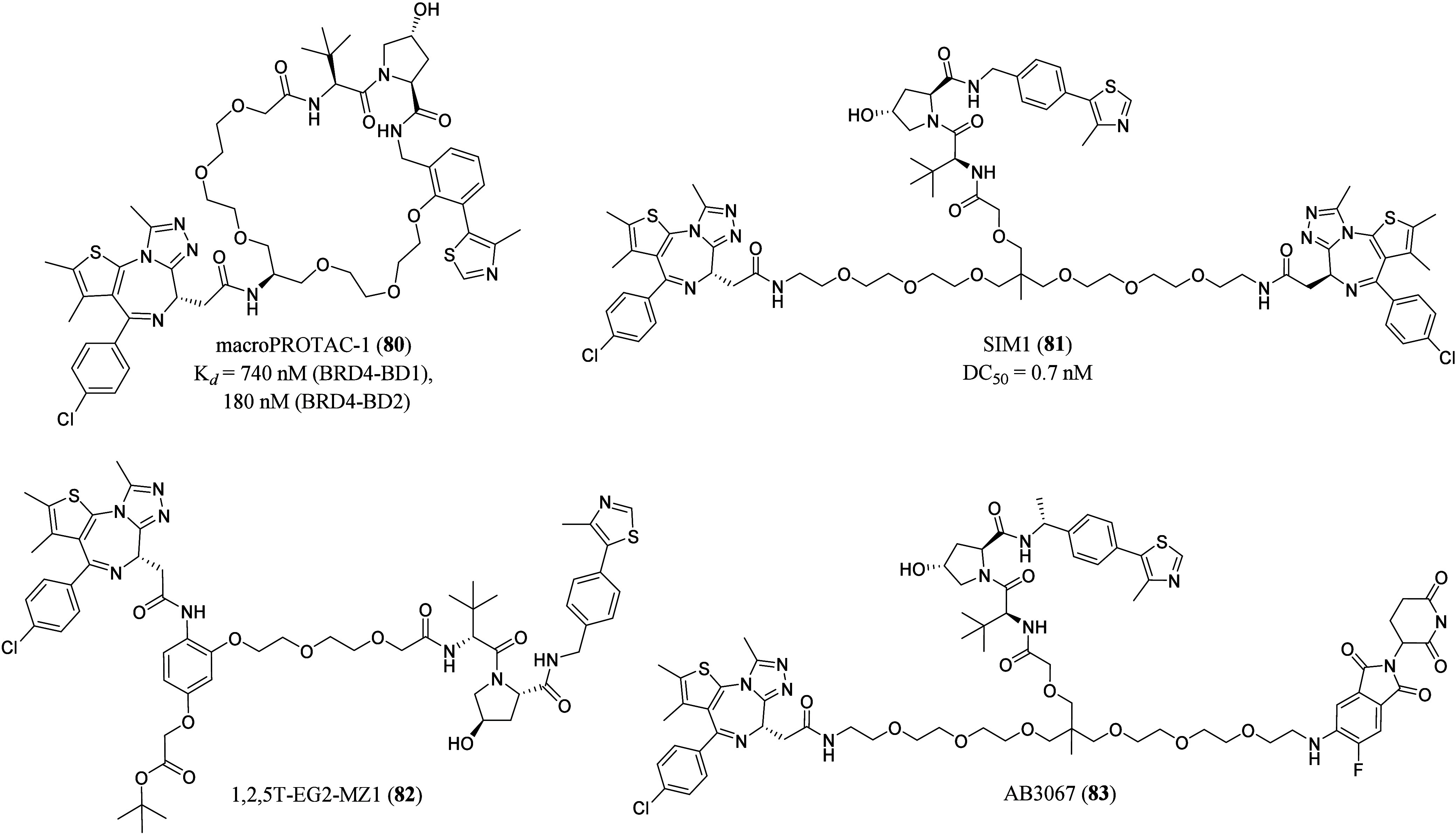
Macrocyclic PROTAC (**80**), which consists of MZ1 (**48**) cyclized with a PEG linker and trivalent PROTACs SIM1 (**81**), 1,2,5T-EG2-MZ1 (**82**), and AB3067 (**83**), which have extra moieties installed for improved potency or further functionalization.

### Trivalent PROTACs

The first trivalent PROTAC SIM1 (**81**) (Figure 6), consisting of two (+)-JQ1 (**4**) molecules and VHL032 tethered together via a branched linker was shown to be more selective to BRD2 at low picomolar with higher degradation efficacy and potency compared to parent bivalent molecule MT1 (**15**).^152^ Mechanistically, **81** was found to simultaneously engage with high avidity to both BDs in a *cis* intramolecular fashion, forming a 1:1:1 ternary complex with VHL and exhibiting positive cooperativity and high cellular stability with prolonged residence time.^152^ Utilizing trivalent CRBN-based and VHL-based PROTACs with different linker lengths, it was demonstrated that efficient protein degradation is depends highly on structural, thermodynamic and kinetic favorability of ternary complex formation.^152^ These were determined by neo-interactions within the ternary complex, including between E3 ligase and target protein to achieve stable, cooperative and long-lived complexes that drove efficient catalytic CRBN-based and VHL-based PROTACs ubiquitination.^152^ On the other hand, compounds that are unable to achieve such favorable ternary complex will be limited by the “hook effect”.^152^ Trivalent PROTAC **82** displayed superior activity compared to parent bivalent inhibitor MT1 (**15**) and PROTAC MZ1 (**48**) in cellular assays, suggesting that trivalent PROTACs might be appropriate for *in vivo* usage despite increased molecular weight and reduction in permeability.^152^

Trivalent PROTAC 1,2,5T-EG2-MZ1 (**83**) (Figure 6), has a *tert*-butyl ester substituted benzene moiety in the linker to allow further synthetic functionalization such as attaching an additional tag (e.g., fluorophore, radioisotope etc.).^153^ The trivalent PROTACs containing a *tert*-butyl ester at different positions were designed based on the crystal structure (PDB #5T35) with MZ1 (**48**).^153^ Among them, **83** with the *tert*-butyl ester group located at the C5 position on the benzene ring, exhibited the best degradation potency and dose-dependent degradation of BRD4 in fibrosarcoma HT1080 cells, and the hook effect was only observed at concentrations above 3 μM.^153^

Most recently, a novel heterotrivalent dual ligase recruiting PROTAC AB3067 (**84**) (Figure 6) was developed to overcome resistance mechanism due to loss of E3 ligase functionality.^154^ By tethering (+)-JQ1 (**4**) with both VHL032 and thalidomide via a PEG linker, **84** displayed targeting of BET proteins and recruitment of both CRBN and VHL ligases with efficient degradation of BRD4 at *D*_max 50_ of 0.6 nM in HiBiT-BRD4 CRISPR knock in HEK293 cells.^154^ Involvement of both CRBN and VHL ligases in the degradation activity of the BRD4 was further confirmed with CRBN and VHL knockout HEK293 cells, which displayed *D*_max50_ of 38 nM and 23 nM, respectively, highlighting the success in recruitment of both E3 ligases with the novel heterotrivalent PROTAC.^154^

### Photocaged PROTACs (*pc*PROTACs)

Photocaged PROTACs (*pc*PROTACs) deploy photocleavable groups to initially mask bioactivity, which will then be optically removed when triggered by light to release the active PROTAC.^155^ This approach allows optical control of the degradation of protein targets with spatiotemporal precision.^155^ The photocleavable groups, also referred to as the “cage”, will be released after light activation and can be attached on different positions of PROTACs, such as the protein–ligand, linker or E3 ligase recruiter.^155^

Light-tunable *pc*PROTAC **84** (Table 6, Section A), based on the chemical structure of MZ1 (**48**), was developed to enable specific intracellular release of PROTAC **85** using light by attaching the photocleavable 4,5-dimethoxy-2-nitrobenzyl (DMNB) group to E3 ligase recruiter VHL032.^156^ Cleavage of the DMNB caging group was achieved with a 365 nm 25 mW light-emitting diode upon irradiation for 180 s, releasing active PROTAC **85** with over 50% conversion within 60 s.^156^ Moreover, **84** displayed stability in cellular environment and no degradation of BRD4 was observed in nonirradiated cells after a 24 h incubation period.^156^

**Table 6:**
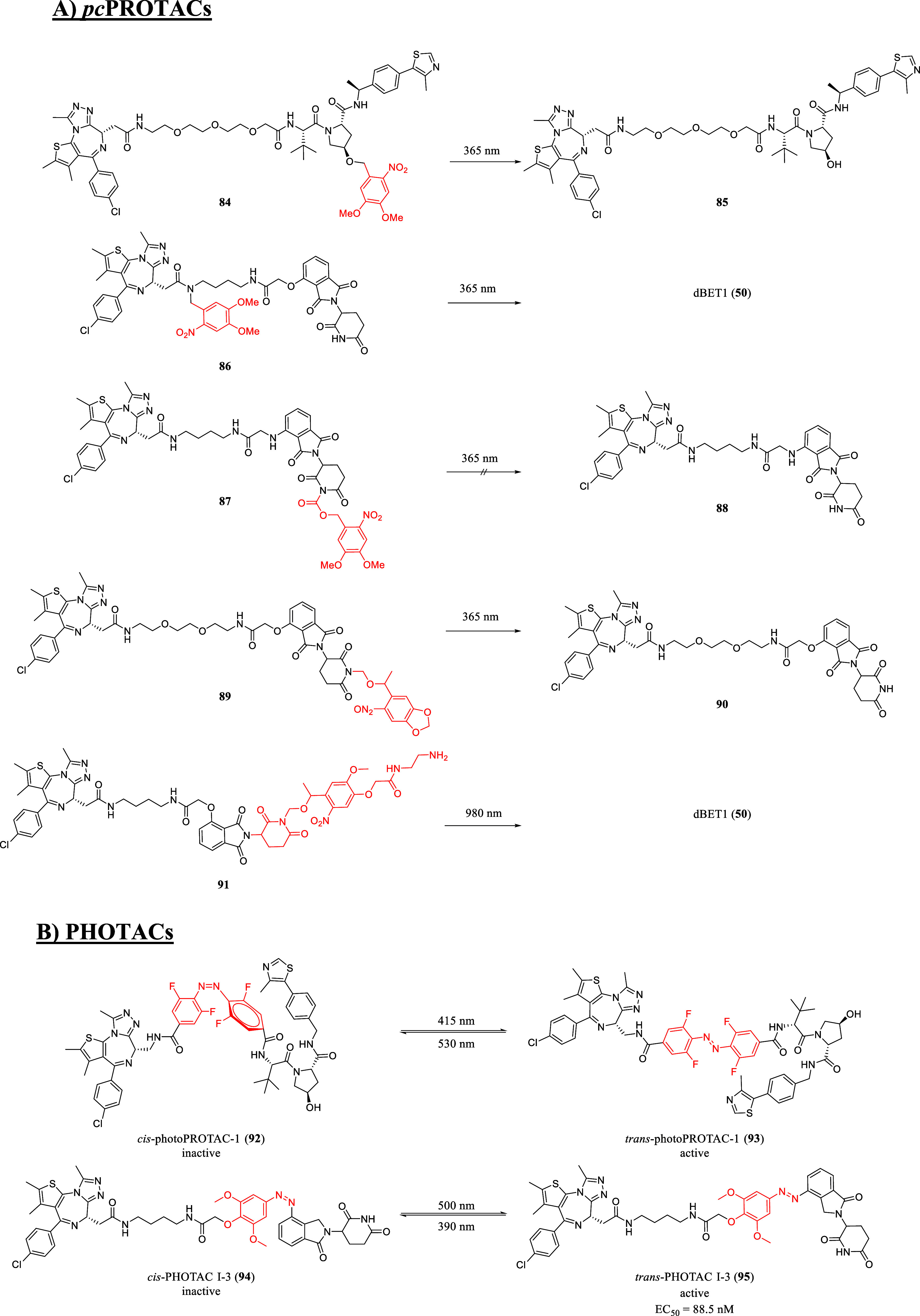

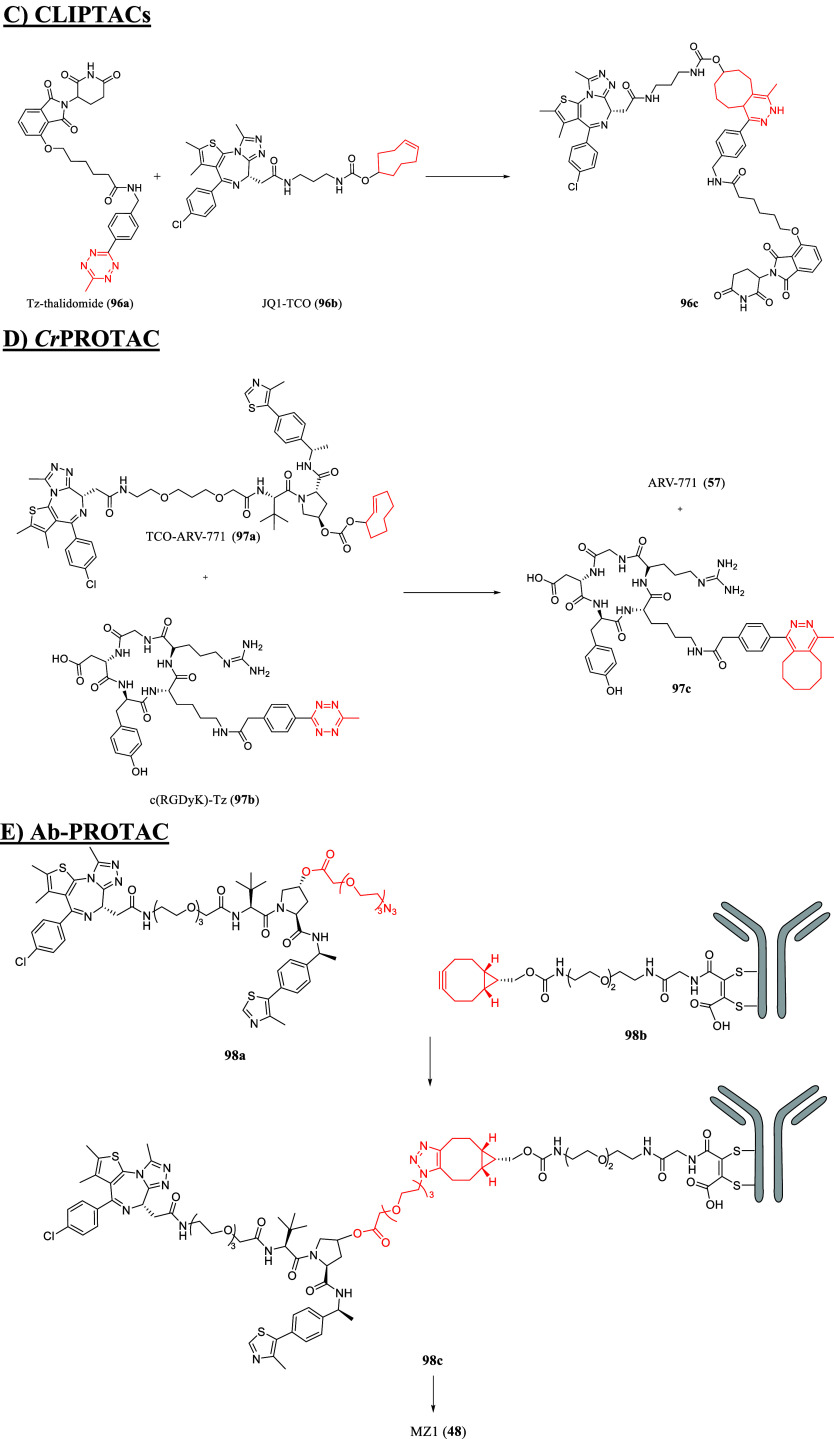

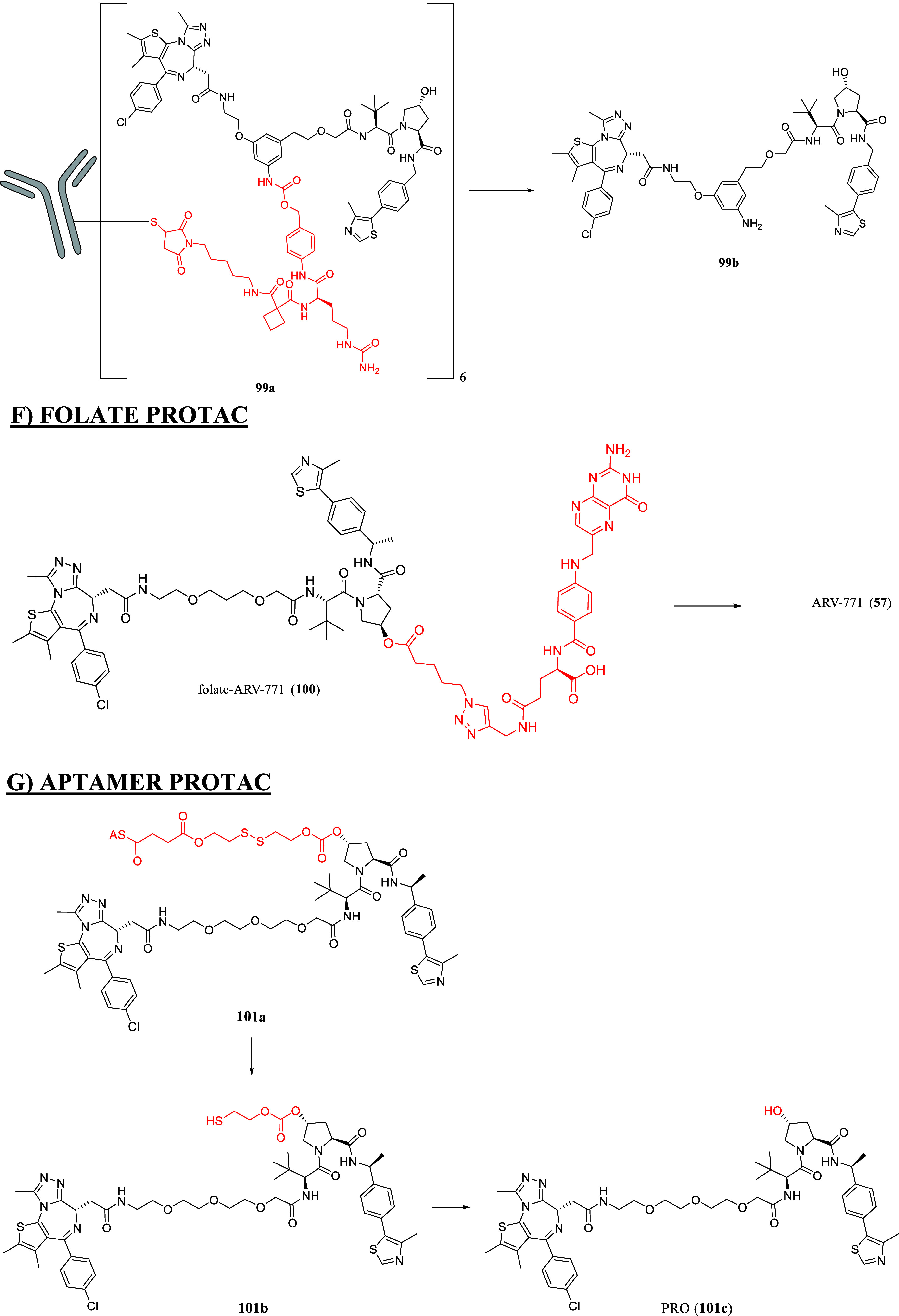

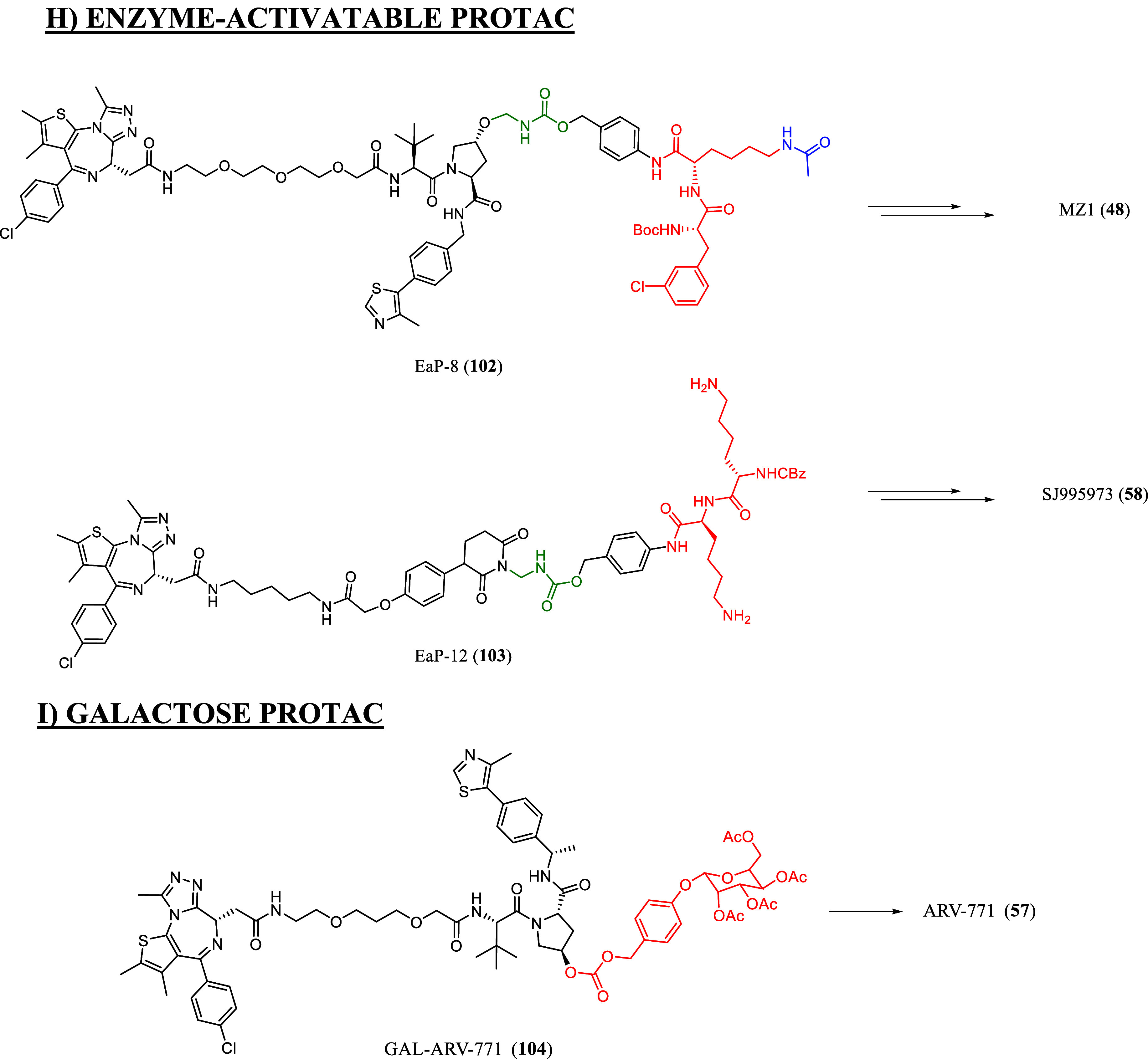
List of JQ1-Derivatized Activatable PROTACs via Different Activation Mechanisms^a^

a (A) DMNB-caged *pc*PROTACs **84** and **86**, which released active PROTACs **85** and dBET1 (**50**) respectively, upon UV-irradiation. *pc*PROTAC **87** did not release **88** upon 365 nm UV-irradiation, while NPOM-caged *pc*PROTAC **89** successfully released **90** upon UV-irradiation. NIR light-activatable photocaged-PROTAC (phoBET1) (**91**) was loaded in UCNPs-based mesoporous silica nanoparticles (UMSNs), that could be activated with NIR light (980 nm) irradiation to release active dBET1 (**50**). (B) Inactive PHOTACs **92** and **94** were converted to respective active PHOTACs **93** and **95** upon irradiation with 415 and 500 nm light, respectively. Conversion of **93** and **95** back to inactive conformation **92** and **94** can be initiated by UV-irradiation at 530 and 390 nm, respectively. (C) CLIPTAC precursors Tz-thalidomide **96a** and JQ1-TCO **96b** reacted intracellularly via copper-free click chemistry to give PROTAC **96c**. (D) Inactive *cr*PROTAC pro-drug, TCO-ARV-771 (**97a**), undergoing bioothogonal click reaction with tetrazine (Tz) modified RGD peptide, *c*(RGDyK)-Tz (**97b**), in the presence of integrin αvβ3 to release active PROTAC ARV-771 (**57**) in cancer cells. (E) Inactive azide precursor **98a** reacted with TCO precursor **98b** via SPAAC click chemistry to form Ab-PROTAC **98c**, which released active PROTAC MZ1 (**48**) in the presence of HER2 positive cancer cells. Selective Ab-PROTAC **99a**, containing a 1,3,5-substituted benzene ring within the linker for conjugation to STEAP1 antibody conjugate via cleavable linker. PROTAC **99b** was released in the presence of STEAP1 expressing cancer cells, and resulted in the targeted degradation of BRD4. (F) Inactive folate-ARV-771 (**100**) was hydrolyzed in the presence of FRα to release active ARV-771 (**57**) in tumor cells. (G) APC **101a** which released **101b** in the presence of GSH in cancer cells, followed by nucleophilic substitution to release ARV-771 (**57**). (H) Dual enzyme activatable PROTAC **102** and monoenzyme activatable PROTAC **103** which released MZ1 (**48**) and SJ995973 (**58**) respectively, in the presence of HDACs and CTSB enzymes in malignant cancer cells. (I) Gal-ARV-771 (**104**) activated in SA-β-Gal-expressed cancer senescent cells to release protein degrader ARV-771 (**57**).

Similarly, using DMNB, various CRBN-targeting *pc*PROTACs **86** and **87** (Table 6, Section A) were developed, with **86** having the DMNB group attached on the linker of dBET1 (**50**), while **87** had the DMNB group attached to the pomalidomide E3 ligase recruiter of the *pc*PROTAC.^157^ Interestingly, only **86** successfully released the active dBET1 (**50**) upon UV irradiation, while no desired PROTAC **88** (Table 6, Section A) was released by **87**.^157^ The effect of **86** was demonstrated in zebrafish, injected with green fluorescent protein (GFP) tagged BRD4 protein, which showed a reduction in fluorescence upon treatment with irradiated **86**.^157^

CRBN-targeting *pc*PROTAC **89** (Table 6, Section A) containing 6-nitropiperonyloxymethyl (NPOM) as the “cage” group attached to the E3 ligase recruiter thalidomide, efficiently degraded GFP-BRD4 in HEK293T cells.^158^ This demonstrated NPOMs ability to release PROTAC **90** (Figure 6, Section A) upon UV-irradiation of **89**.^158^

UMSNs@phoBET1, a near-infrared (NIR) light-activatable *pc*PROTAC phoBET1 (**91**) (Table 6, Section A), loaded in upconverting nanoparticles-based mesoporous silica nanoparticles (UMSNs), could be activated with NIR light (980 nm) irradiation to release active dBET1 (**50**), inducing MV4-11 cancer cell apoptosis.^159^ UMSNs act as a NIR to UV converter to transfer the excitation wavelength to the biological window of NIR, allowing UMSNs@phoBET1 to undergo upconversion luminescence-induced photolysis reaction, remove the caged group and release active dBET1 (**50**), providing a strategy for spatiotemporal activation of *pc*PROTACs by NIR light.^159^ *In vitro* and *in vivo* experiments verified that UMSNs@phoBET1 was able to control the degradation of BRD4 with NIR light irradiation and effectively suppress tumor growth.^159^ This NIR light-activatable *pc*PROTAC methodology demonstrated success in overcoming the negative effect of short-wavelength light-controlled *pc*PROTACs and presents a new opportunity for precise regulation of PROTACs in living tissues.^159^

### Photochemically Targeting Chimeras (PHOTACs)

Photochemically targeting chimeras (PHOTACs) are photoswitchable PROTACs that can be initiated with the spatiotemporal precision of light.^160^ These molecules, consist of a linker, a ligand targeting an E3 ligase, a photoswitch, and a ligand recruiting a protein target.^160^ By installing a light-sensitive conformationally altered groups in PROTAC, at either the linker or E3 ubiquitin ligase ligand, PHOTACs are able to regulate protein degradation reversibly.^161–163^ This novel concept was introduced in PROTAC ARV-771 (**57**), in which the PEG linker of **57** was replaced with an azobenzene linker.^161,162^ By introducing 415 nm light irradiation on PHOTOPROTAC-1 (**92**) (Table 6, Section B), azobenzene was converted from the inactive *cis*-conformation to the active *trans-*conformation **93**, giving a distance of 11 Å between the two amide bonds, which was consistent with the original ARV-771 (**57**) linker, initiating targeted protein degradation.^161,162^ On the other hand, light irradiation at 530 nm gave the azobenzene in *cis-*conformation, with a nonideal 8 Å distance between the two amide bonds, the protein target was thus not ubiquitinated and degraded.^161,162^ Similarly, utilizing an azobenzene linker, inactive PHOTAC I-3 (**9**) (Table 6, Section B) was able to show degradation when applying 500 nm wavelength light irradiation, converting it to its active *trans*-conformation **95**.^160^ These studies demonstrated the reliability of azobenzene as a photoswitch to introduce spatiotemporal precision in PHOTACs and potential applications in photomedicine.^160^

### Clickable Degraders

Click chemistry has gained significance supporting the quick and efficient manner to synthesis molecules, and in bioorthogonal chemistry enabling novel biological studies in living systems.^164^ It is not surprising that several groups have applied click and bioorthogonal chemistry strategies to develop novel degrader tools. Several reviews have already extensively summarized this work,^82,165,166^ hence, this review will only highlight a number of novel JQ1-derivatized degraders that utilized click chemistry in their methodologies.

### In-Cell Click-Formed Proteolysis Targeting Chimeras (CLIPTACs)

In-cell click-formed proteolysis targeting chimeras (CLIPTACs) were reported to form PROTAC intracellularly via bioorthogonal click reaction of two precursors that are smaller in size.^167^ Tz-thalidomide (**96a**), a tetrazine tagged thalidomide derivative reacted rapidly in cell with JQ1-TCO (**96b**) to form a CRBN E3 ligase recruiting PROTAC **96c** (Table 6, Section C), which successfully degraded BRD4.^25,167^ Control experiments were conducted to show that degradation was only initiated upon addition of the two precursors, while addition of only one precursors or the clicked PROTAC **96** showed no degradation in live cells.^167^ This highlights the advantages of utilizing smaller CLIPTAC molecules to overcome the challenges of cellular permeability of large PROTAC molecules.^167^

### Click-Release PROTAC (*cr*PROTACs)

Click-release PROTACs (*cr*PROTACs) have been developed to overcome the potential toxicity and side effects of PROTACs due to uncontrolled degradation of proteins and undesirable ligase-mediated off-target effects.^168^ Precise and selective degradation activity was demonstrated by *cr*PROTACs utilizing a bioorthogonal on-demand prodrug strategy for cancers.^168^ Inactive *cr*PROTAC TCO-ARV-771 (**97a**) (Table 6, Section D), consisting of PROTAC ARV-771 (**57**) functionalized with a TCO moiety, was able to react with a tetrazine (Tz) modified arginylglycylaspartic acid (RGD) peptide, *c*(RGDyK)-Tz (**97b**), to release the active ARV-771 (**57**).^168^ The bioorthogonal reaction is initiated by the integrin αvβ3 biomarker in cancer cells enabling cancer cells specific targeted degradation of BRD4.^168^ This work demonstrated that *cr*PROTAC prodrugs can be selectively activated in an integrin αvβ3-dependent manner to produce active PROTACs that degrade target proteins in cancer cells, potentially reducing side effects observed due to targeting healthy cells.^168^

Ab-PROTACs, folate-PROTACs, enzyme-activatable PROTACs, galacto-modified PROTACs and aptamer PROTACs (APCs), have been developed to overcome the nonspecificity of conventional PROTACs. By addition of a biologically recognizable moiety that is specific to cancer cells, these PROTACs possessed cancer-targeting properties, allowing selectivity to cancer cells, overcoming potential side-effects due to targeting of noncancerous cells.

### Antibody-PROTAC (Ab-PROTAC) Conjugates

The idea of conjugating antibody–drug conjugates to PROTACs in order to incur tissue selectivity was demonstrated in antibody PROTAC (Ab-PROTAC) conjugate (**98c**) (Table 6, Section E).^169^ Trastuzumab, an antibody against human epidermal growth factor receptor 2 (HER2), was conjugated to MZ1 (**48**) at the hydroxyl group of the proline moiety on MZ1 (**48**).^169^ The trastuzumab-facilitated delivery of the PROTAC to HER2 positive cells was achieved with a cleavable ester bond, that allowed MZ1 (**48**) to be released upon ester hydrolysis initiating ubiquitination of the target protein.^169^ Conjugation challenges due to the large molecular size of PROTACs was overcome by using SPAAC to form **98c** with smaller precursors functionalized with an azide (**98a**) and TCO (**98b**) for targeted delivery to HER2 expressing breast cancer cells SK-BR-3 and BT-474.^169^ These cells revealed complete degradation of BRD4 after 4 h treatment with 100 nM of **98c**, while no degradation was detected in HER2 negative cell lines.^169^ This work successfully exhibited the selective delivery of Ab-PROTAC **98c** to HER2 positive cells via conjugation with trastuzumab through recognition of cancer cell-specific antigens, which enabled reduction in toxicity and side effects of the parent MZ1 (**48**).^169^

Different antibody-cleavable linkers (e.g., peptidomimetic, disulfide, maleimide), antibody attachment chemistry, and antibody loading for Ab-PROTACs were investigated and optimized.^170–172^ Ab-PROTAC **99a** (Table 6, Section E) highlights that conjugation to the antibody is also feasible via linker modifications compared to the more commonly utilized hydroxyl group of VHL032.^170–172^ The 1,3,5-substituted benzene ring linker allowed for conjugation with the STEAP family member 1 (STEAP1) antibody via cleavable linkers. Experiments showed release of **99b** resulted in BRD4 degradation in STEAP1 highly expressed PC3-S1 prostate cancer cells with a DC_50_ value of 4.4 nM in 72 h.^171,172^ Future research on Ab-PROTACs is necessary to overcome the immunogenic nature of novel degrader tools.^170^

### Folate PROTACs

Cancer cell selectivity was also achieved by attaching folate to PROTAC ARV-771 (**57**), forming inactive folate-ARV-771 (**100**) (Table 6, Section F), which bound specifically to folate receptor α (FRα), that is overexpressed in several tumor types.^109,173^ Similar to Ab-PROTACs, an ester linkage was used to tether the folate group, forming folate-ARV-771 (**100**), which can be easily hydrolyzed to give active ARV-771 (**57**).^109,173^ On the other hand, amide linkage was not able to give the desired ARV-771 (**57**) in the cell as it was less susceptible to hydrolysis.^173^ Folate-ARV-771 (**100**) displayed comparable potencies with ARV-771 (**57**) in FRα-expressing cancer cell lines, including HeLa (246 nM vs 183 nM), ovarian cancer cells OVCAR8 (297 nM vs 215 nM), and the human breast ductal carcinoma cells T47D (18 nM vs 13 nM).^173^ In contrast, **100** was much less efficient than ARV-771 (**57**) in noncancerous cell lines, such as fibroblast cells HFF-1 and 3T3, and proximal tubule epithelial HK2 cells.^173^ This work demonstrated the feasibility of using folate-PROTACs to improve the selectivity of PROTACs and limit its toxicity on noncancerous cells.^173^ Nonetheless, the bulky folate group posed permeability and pharmacokinetics issues, which should be further looked into.

### Aptamer PROTACs (APCs)

Aptamer PROTACs (APCs) were developed to overcome the nonspecific nature of conventional PROTACs and disadvantages of Ab–PROTACs conjugates, such as controlling the administration dose, complex pharmacokinetics and immunogenicity.^174^ Aptamers are single-stranded nucleic acids that exhibit complex three-dimensional structures, which bind to the protein target with high specificity and affinity through chemical bonds (such as stems, rings, hairpins, and G4 polymers).^174,175^ In APC **101a**, aptamer AS1411 (AS) was conjugated to PRO (**101c**) as it specifically recognizes and binds to nucleolin, which is highly expressed on tumor cell membranes.^174,176^ APC **101a** works via a two-stage process (Table 6, Section G): 1) the disulfide bond within the linker of APC **101a** will be broken via nucleophilic attack by endogenous glutathione in tumor cells; 2) the newly formed mercapto group in **101b** can then attack the carbon anhydride ester bond to release the active PROTAC **101c**.^174^ They successfully demonstrated APC’s BRD4 degradation in a concentration-dependent manner with DC_50_ of 22 nM and possessed antiproliferative activity against MCF-7 and MCF-10A breast cancer cells with an IC_50_ = 56.9 nM and 3.13 μM, respectively.^174^

### Enzyme-Activatable PROTACs

Selective, (+)-JQ1 (**4**)-derived enzyme-activatable PROTACs, utilized enzyme-recognition moieties to enable cell-selective protein degradation when activated by specific elevated enzymes in cancer cells.^177^ Methylene alkoxy carbamate (MAC) was installed as an optimal self-immolative linker with high stability and release efficiency for conjugating enzyme-recognition moieties in VHL and CRBN targeting PROTACs, EaP-8 (**102**), and EaP-12 (**103**), respectively, (Table 6, Section H).^177^ The dual-enzyme-activatable BRD4 degrader EaP-8 (**102**), caged with HDACs and Cathepsin B (CTSB)-targeting ligands, required the presence of two cancer-associated enzymes for activation by removal of the ε-*N*-acetyl group by HDACs, followed by removal of the lysine residue, which was recognized and cleaved by CTSB to initiate the sequential elimination and liberation of MZ1 (**48**).^177^ EaP-8 (**102**) demonstrated comparable efficiencies as MZ1 (**48**) in inducing BRD4 degradation and suppressing cell growth (IC_50_ = 476 nM) in malignant colon cancer cells HCT116, while being less efficacious in nonmalignant Caco-2 cells with IC_50_ = 10.5 μM, while MZ1 (**48**) displayed no differential cytotoxicity between both cell lines.^177^ Moreover, EaP-8 (**102**) efficiently induced BRD4 degradation and inhibited cell growth in HT-29 colorectal cancer cells and HepG2 liver cancer cells, both of which are malignant cancer cells having elevated activities of HDACs and CSTB.^177^ These results demonstrated that **102** exhibited a high degree of selectivity for malignant cancer cells with increased HDACs and CTSB activities over nonmalignant cells in mediating BRD4 degradation and cell death.^177^

Monoenzyme activatable PROTAC EaP-12 (**103**) (Table 6, Section H), incorporated a highly specific CTSB-recognition lysine–lysine dipeptide substrate into the phenyl-glutarimide moiety of SJ995973 (**58**) via the MAC linker for CTSB activation.^177^ EaP-12 (**103**), like SJ995973 (**58**), degraded BRD4 in malignant colon cancer HCT116 cells more efficiently than in nonmalignant Caco-2 cells with an IC_50_ = 927 nM and 11.4 μM, respectively.^177^ Moreover, the IC_50_ value of **103** was much higher than that of parental PROTAC **58** in Caco-2 cells (IC_50_ = 1.01 μM), displaying the superiority of EaP-12 (**103**) in differentiating malignant cancer cells from nonmalignant cells to induce selective BRD4 degradation.^177^ This work successfully demonstrated the application of self-immolative MAC linkers and enzyme-recognition moieties that can be applied in developing enzyme-activatable VHL and CRBN-based PROTACs for targeted cancer treatment.^177^

### Galacto-Modified PROTACs (GAL-PROTACs)

Galacto-modified PROTACs (GAL-PROTACs) utilizes a senescence-associated β-galactosidase (SA-β-Gal)-responsive prodrug strategy for selective degradation of senescent cancer cells.^178^ The prodrug Gal-ARV-771 (**104**) (Table 6, Section I) composing of SA-β-gal substrate galactose and PROTAC ARV-771 (**57**) was selectively activated in SA-β-Gal-expressed cancer senescent cells to release active ARV-771 (**57**) and was found to display senolytic indexes higher than the parent compound ARV-771 (**57**).^178^ Discriminative degradation was observed between normal A549 (n-A549) lung cancer cells and etoposide-induced senescent A549 (s-A549) cells during treatment with **104** (100 nM), while parent ARV-771 (**57**) displayed no differences in both normal and senescent A549 cells.^178^ Gal-PROTACs serve as a novel and powerful tool for the selective elimination of relapse-driving senescent cancer cells as well as mitigation of other human senescence-related disorders.^178^

### Regulated Induced Proximity Targeting Chimera (RIPTAC)

A new class of heterobifunctional molecules to target and eliminate cancer cells while leaving healthy cells unharmed, functioned by formation of a stable ternary complex with two proteins, one specifically found in cancer cells (target protein) and an effector protein (EP) in healthy cells, which is a pan-expressed protein that is essential for cell survival, RIPTACs selectively disrupt the function of the EP and cause apoptosis of cancer cells.^179–181^ RIPTAC **105** (Figure 7) was tested in HEK293-derived cell line with overexpression of FLAG-tagged HaloTag7-FKBP^F36V^ using lentivirus-“293-HFL” (HaloTag-FKBP-Lentivirus) cells, and a cell line overexpressing only EGFP that was generated as a control-“293-GFPL” (GFP-Lentivirus) cells.^180^ **105** gave EC_50_ of 192 nM in 293-HFL cells and 11.4 μM in 293-GFPL, validating the discriminative targeting of cancer cells with this novel modality.^180^

**Figure 7:**
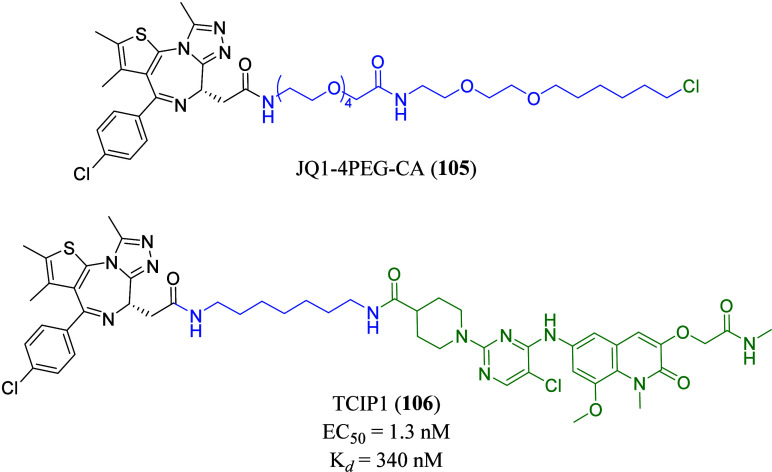
Novel proximity-inducing modalities RIPTAC (**105**) and TCIP1 (**106**), which selectively target cancer cells by bringing BRD4 in close proximity to effector protein and BCL6, respectively.

### Transcriptional/Epigenetic Chemical Inducers of Proximity (TCIPs)

Transcriptional/epigenetic chemical inducers of proximity (TCIPs) recruit an endogenous cancer driver, or a downstream transcription factor, to the promoters of cell death genes, leading to activation of their expression.^182^ TCIPs produce their effect by activating cell death signaling and rewiring cancer driver molecules to drive the phenotype.^182^ TCIP1 (**106**) (Figure 7) consisting of (+)-JQ1 (**4**) linked to a B cell lymphoma 6 (BCL6) binder BI-3812, increased binding of BRD4 by 50% over genomic BCL6-binding sites to produce transcriptional elongation at pro-apoptotic target genes, while reducing binding of BRD4 over enhancers by only 10%, reflecting a gain-of-function mechanism.^182^ **106** also selectively killed with an EC_50_ of 1.3 nM against B-cell non-Hodgkin lymphoma KARPAS422 cell.^182^ **106** induced a stable, cooperative protein–protein interaction between BRD4­(BD1) and the BTB domain of BCL6, allowing intracellular ternary complex formation with *K*_*d*_ of 340 nM in TR-FRET assays.^182^ Inducing G1/S and G2/M block in the cell cycle, **106** repressed MYC and its targets while activating pro-apoptotic genes in KARPAS422 cells.^182^ **106** also killed chemotherapy-resistant diffuse large B cell lymphoma cell lines, *TP53* mutant lines, at EC_50_ of 1–10 nM in 72 h and exhibited cell-specific and tissue-specific effects.^182^ This approach suggests opportunities to use diverse cancer drivers to kill cancer cells by rewiring the cancer driver circuitry.^182^

### “Bump and Hole” Engineering and Bromotags

A “bump-and-hole” approach was developed to engineer selectivity onto small-molecule modulation of BD. As a chemical genetic approach, it allows systematic generation of an orthogonal high-affinity protein–ligand variant pair based on engineered shape complementarity between the BD and a small-molecule inhibitor, by removing a bulky gatekeeper residue.^183,184^ The “hole”-modified protein target can be specifically sensitized to a designed small-molecule inhibitor with a complementary “bump”, resulting in an isoform-specific perturbation of the protein while having wild type characteristics.^184^ Derivatives of I-BET762 (**5**) (Table 1), ME (**107**) and ET (**108**) (Figure 9), were designed based on crystal structure analysis which suggested functionalization of the side chain methylene of **5** might provide a desired bump to fill the hole introduced by mutation of the leucine residue (L94) in BRD4 from the ZA loop to alanine.^183–185^ Utilizing biophysical studies, this novel concept of orthogonal BD:ligand pairs were proven to be highly selective and capable of dissecting the role of individual BDs of BRD4 in chromatin binding, opening up a novel approach of implementing selective chemical control.^183,184^ Despite attempts to improve the metabolic stability of ligands **107** and **108** by replacing the methoxy with an amide group, binding affinities were found to be negatively impacted.^186^ Further optimization of the “bump-and-hole” approach with the orthogonal BD:ligand pairing was reported by using a less disruptive leucine/valine mutation and ligands **109** and **110**.^184–188^ A more efficient and cost-effective synthetic route was also developed to synthesize structurally diverse and enantiopure triazolodiazepines, which were later applied in this novel “bump- and-hole” studies.^187^

The concept of BromoTag is a complementary strategy that involves modifying the gene which encodes for the protein target by adding a tag, also called the “degron tag”.^189,190^ This strategy allows a BromoTag to bind with an orthogonal PROTAC with high affinity, directly recruiting an E3 ligase for ubiquitination and promoting degradation of the protein target (Figure 8),^189,190^ enabling the study of proteins that lack known selective ligands (peptides or small molecules).^189,190^ Genetically modified BD BRD4^BD2 L387A^ was expressed to form a fusion protein via transgene expression or CRISPR-mediated locus-specific knock-in, while its orthogonal BromoTag AGB1 (**111**) (Figure 8), which links the “bump-and-hole” ligand ET-JQ1-OMe (**110**) with VHL-032, demonstrated a strong, cooperative ternary complex between the E3 ligase and the fusion protein, in an efficient manner.^189^

**Figure 8:**
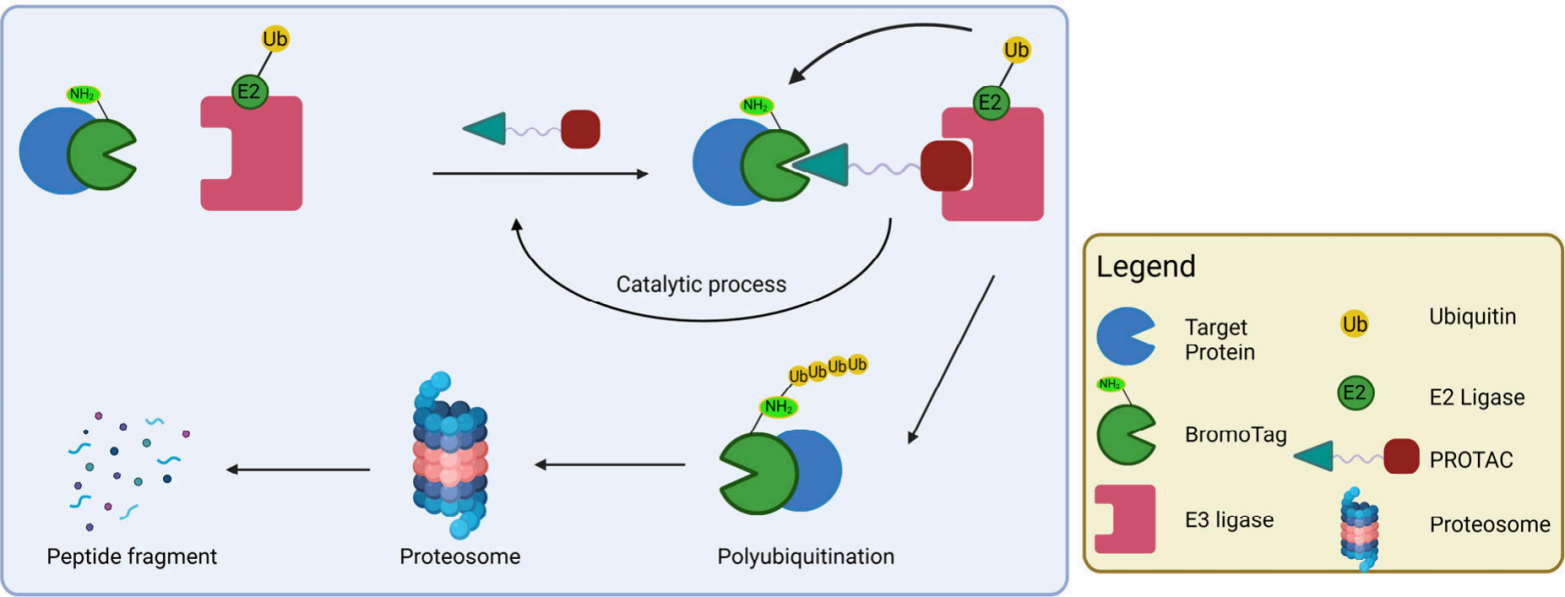
Schematic representation of the mechanism of the BromoTag degron system. The BromoTag degron system utilizes the “bump and hole” concepts on PROTACs, where the protein was modified to bind orthogonally to matching ligand, allowing improved binding specificity.

Similarly compounds XY-06-007 (**112**) and XY-06-011 (**113**) (Figure 9), which target E3 ligase CRBN, were demonstrated to bind to BRD4^(BD1)L94V^ with increased selectivity over the wild-type BD, which translated into potent and preferential degradation of the mutant BRD4^(BD1)L94V^.^190^ Showing proteome-wide selectivity and *in vitro* mouse hepatic microsome stability, **112** and **113** were identified as suitable candidates for future *in vivo* studies.^190^

**Figure 9:**
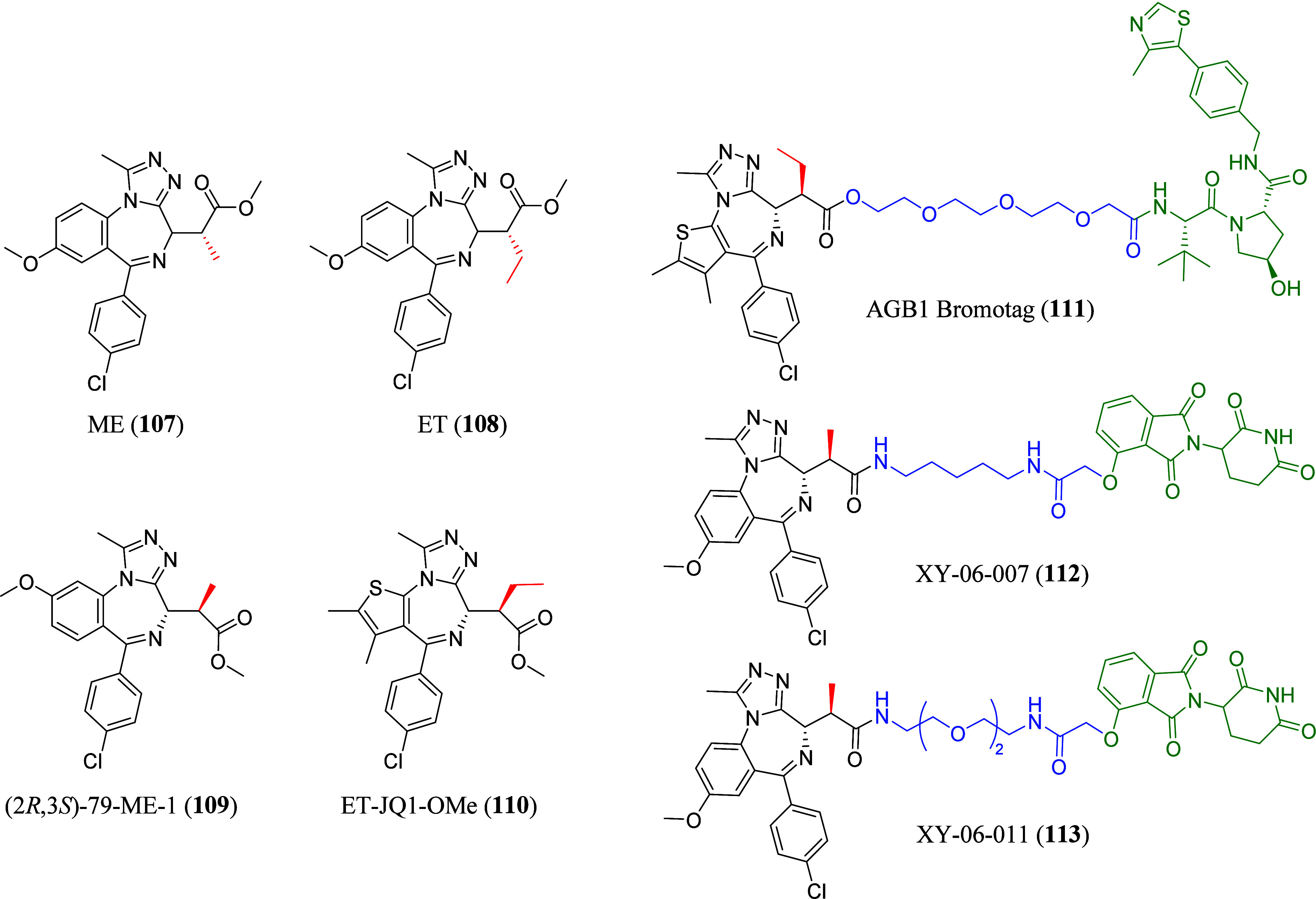
“Bump- and-hole” ligands **107**–**110**, which possess an orthogonal structure to a genetic modified BRD4 protein for improved selectivity and binding. BromoTag probes modified with ethyl and methyl groups in **111**–**113** for orthogonal BD:ligand pairing were used with modified BRD4 protein for improved binding specificity.

## Conclusion

Thienotriazolodiazepine-derivatives have proven to be high-quality probes. The most notable molecule being (+)-JQ1 (**4**), fulfilling key criteria for chemical probes, including an IC_50_ or *K*_d_ < 100 nM in biochemical assays, an EC_50_ < 1 μM in cellular assays, > 30-fold selectivity within the protein target family, in addition to extensive profiling of off-targets outside the protein target family, and a well validated negative control (−)-JQ1. Furthermore, the discovery of (+)-JQ1 (**4**) has opened a myriad of possibilities for the treatment of cancers via inhibition of BET proteins. The molecule also provided a valuable tool to investigate downstream effect on MYC, a protein that previously was difficult to perturb.

By labeling (+)-JQ1­(**4**) and related thienotriazolodiazepine analogs with different reporter tags, parallel studies developing different concepts advanced our knowledge on the functions of BET proteins. Due to the well-established SAR and rigorous pharmacological evaluation, (+)-JQ1­(**4**) and BET proteins have become an ideal model system for testing novel chemical modalities and drug discovery concepts. This is illustrated by the breadth of (+)-JQ1 (**4**) chemical probes, including PET radiotracers and fluorescent, covalent, and protein degrader probes that have been developed to target BET proteins. Arguably, (+)-JQ1-based PROTACs, have been the most impactful (+)-JQ1 (**4**) chemical probes, as the development of “drug-like” PROTACs has been without doubt instrumental for the expansion of the field of PROTACs and other protein degraders beyond academia. It is encouraging that the progress in (+)-JQ1-based PROTAC development has contributed to the advancement of novel treatments for oncological diseases such as degraders ARV825 (**56**) and ARV771 (**57**), which are both currently in clinical trials. If successful, this would be a breakthrough for both PROTAC and BET protein–drug discovery.

The development of SNIPERs, Ab-PROTACs, folate-PROTACs and enzyme-activating PROTACs is beneficial in overcoming the toxicity issues induced by many PROTACs in clinical trial. Nonetheless, their large molecular size beyond the traditional medicinal chemistry “rule of five” could make their bioavailability and pharmacokinetics more complicated than for conventional PROTACs. With the help of formulation scientists, some of these issues associated with new degrader modalities might be overcome, which again emphasizes the importance of collaboration between experts from different scientific fields.

The work on BacPROTACs successfully demonstrated the feasibility of applying a targeted protein degradation mechanism on bacteria and opened up the potential of developing antimicrobials with a novel mechanism. If successful, this also serve as a stepping stone toward the utilization of targeted protein degradation on various “host–pathogen” caused diseases, which are still in need of better therapeutics.

Photoaffinity binding tools, which are beneficial for on-target covalent binding for further analysis, are helpful for target deconvolution studies. However, the usage of harmful UV-light makes it challenging to be applied in live cells and living animals, and results obtained using such harsh treatment may result in data that are compromised due to the stress induced on living systems. Nonetheless, photoaffinity tools that bind to protein in a covalent and irreversible manner, have proven to be advantageous for a range of *in vitro* studies. The development of alternative labeling tools and concepts, such as the electroaffinity and proximity labeling probes and approaches, are overcoming some of the challenges of photoaffinity labeling while also providing new opportunities for target identification and validation.

Moreover, the significance of sharing (+)-JQ1 (**4**) and MZ1 (**48**) among the scientific community has proven to be beneficial for breakthroughs in the study of BET proteins. This model of open access to chemical probes within the scientific community has led to fast progress in method development, target interrogation, and developed therapeutic products that will benefit patients. The growing trend of organizations and consortiums that commit to such open research platforms, such as opnME by Boehringer Ingelheim and the Structural Genomic Consortium (SGC), which distributed chemical probes from various pharmaceutical companies free of charge, is encouraging.

Despite, enormous progress particularly in the past decade, further knowledge gain on BET proteins’ and their role in biological systems remains crucial for drug discovery. The development of quality probes and novel concepts should constantly be explored to advance therapeutics for diseases that remain poorly treated or are untreatable today.

## Abbreviations

ABPP: Activity-based protein profiling
Ab-PROTAC: Antibody proteolysis targeting chimeras
ACT: Aryl-5-carboxytetrazole
AhR: Aryl hydrocarbon receptor
APC: Aptamer proteolysis targeting chimeras
ATAD5: ATPase family AAA domain containing 5
ATTEC: Autophagosome-tethering compound
ALL: Acute lymphoblastic leukemia
AUTAC: Autophagy-targeting chimera
BacPROTAC: Bacterial PROTAC
BET: Bromodomain and extra-terminal
BBB: Blood–brain barrier
BCL6: B cell lymphoma 6
BD: Bromodomain
BioTAC: Biotin targeting chimera
BRD2: Bromodomain-containing protein 2
BRD3: Bromodomain-containing protein 3
BRD4: Bromodomain-containing protein 4
BRDT: Bromodomain testis associated
ByeTAC: Bypassing E3 targeting chimeras
CACO: Colorectal adenocarcinoma
CDDO: Bardoxolone
cIAP1: Cellular inhibitor of apoptosis protein-1
cIAP2: Cellular inhibitor of apoptosis protein-2
CLIPTAC: In-cell click-formed proteolysis targeting chimeras
ClpCP: Caseinolytic protease proteolytic subunit
CRBN: Cereblon
CRISPR: Clustered regularly interspaced short palindromic repeats
CRPC: Castration-resistant prostate cancer
*cr*PROTAC: Click released PROTAC
CTSB: Cathepsin B
CUL2: Cullin-2
CUL4: Cullin-4
CuAAC: Copper-catalyzed azide–alkyne cycloaddition
DdlA: d-Alanine ligase A
DCAF: DNA damage-binding protein 1 (DDB1)- and Cullin-4A (CUL4)-associated factor
DDB1: DNA damage-binding protein 1
DMNB: 4,5-Dimethoxy-2-nitrobenzyl
DNA: Deoxyribonucleic acid
DZE: Diazetidinone
EZH2: Enhancer of zeste homologue 2
GABA: Gamma-aminobutyric acid
Gal-PROTAC: Galacto-modified PROTAC
GFP: Green fluorescent protein
GSH: Glutathione
HCC: Hepatocellular carcinoma
HDAC: Histone deacetylase
HER2: Human epidermal growth factor receptor 2
HIV: Human immunodeficiency virus
IAP: Inhibitor of apoptosis protein
IBG: Intramolecular bivalent glues
IMiD: Immunomodulatory imide drugs
IP-MS: Immunoprecipitation mass spectrometry
JQ1: (6*S*)-4-(4-Chlorophenyl)-2,3,9-trimethyl-6*H*-thieno­[3,2-*f*]­[1,2,4]­triazolo­[4,3-*a*]­[1,4]­diazepine-6-acetic acid 1,1-dimethylethyl ester
KLHDC2: Kelch domain-containing protein 2
KLHL20: Kelch-like protein 20
LC3: Light chain 3B
MDM2: Mouse double minute 2
MS: Mass spectrometry
MYC: Myelocytomatosis oncogene
NAMPT: Nicotinamide phosphoribosyltransferase
NIR: Near infrared
NMC: NUT-midline carcinoma
PAL: Photoaffinity labeling
*pc*PROTAC: Photocaged proteolysis targeting chimeras
PEG: Polyethylene glycol
PET: Positron emission tomography
PHOTAC: Photochemically targeting chimeras
PL: Proximity labeling
PROTAC: Proteolysis targeting chimeras
RIPTAC: Regulated induced proximity targeting chimera
RNA: Ribonucleic acid
RNF4: Ring finger protein 4
SA-β-Gal: Senescence-associated β-galactosidase
SNIPER: Specific and nongenetic inhibitor of apoptosis protein (IAP)-dependent protein erasers
SPAAC: Strain-promoted azide–alkyne cycloaddition
STEAP1: STEAP family member 1
TCIP: Transcriptional/epigenetic chemical inducers of proximity
TCO: *trans*-Cyclooctene
ThrC: Threonine synthase
TNBC: Triple-negative breast cancer
TR-FRET: Time-resolved Förster resonance energy transfer
UMSN: Upconverting nanoparticles-based mesoporous silica nanoparticles
UPS: Ubiquitin proteasome system
UV: Ultraviolet
VHL: Von Hippel–Lindau
XIAP: X-linked inhibitor of apoptosis protein
